# A retrospective analysis of the tuberculin skin test reactions of a single source population of Mauritian *Macaca fascicularis* held in quarantine during 2017

**DOI:** 10.1371/journal.pone.0265942

**Published:** 2022-04-14

**Authors:** Matthew Panarella, Sharon Hursh

**Affiliations:** Buckshire Corporation, Perkasie, Pennsylvania, United States of America; CEA, FRANCE

## Abstract

A published range of reactions to the tuberculin skin test (TST) using mammalian (human) old tuberculin (MOT) in a population of Mauritius origin *Macaca fascicularis* in US laboratory animal medicine does not exist. The objectives of this report are to quantify the reactions among juvenile, adolescent, and male adult cynomolgus macaques and to provide the laboratory animal medicine practitioner with a reference range of reactions to MOT in Mauritian juvenile macaques. Monkeys in a US foreign animal quarantine must be tested negative for mycobacterial infection including *Mycobacterium tuberculosis* (Mtb) using MOT as required by the CDC Division of Global Migration and Quarantine. The TST relies on visual observation post injection of an individual animal’s reaction or nonreaction. During 2017, 933 Mauritius origin macaques from one supplier were quarantined in nine separate cohorts in our facility. 848 or > 90% of the animals were juveniles between 1.5 to 3 years of age, comprised of 425 males and 423 females. The population also included 46 adolescents (21 males, 25 females) and 39 adult males. A total of 33 TST sets were performed on the nine cohorts, resulting in 3418 individual TSTs being administered. 1595 (46.6%) injections were made in juvenile males and 1529 (44.7%) in juvenile females. A total of 172 reactions to the TST were recorded at the final assessment completed at the 72-hour post injection time point from all animal ages and sexes. 162 reactions (94.2% of all reactions) occurred in juvenile animals, consisting of 96 bruises (59.3% of all juvenile reactions) in males and 58 bruises (35.8% of all juvenile reactions) in females. Bruising was the most common reaction in juvenile animals and in all animals regardless of age or sex. Bruising occurred within all 33 TST sets ranging from a low of 0% to a high of 30% in male juveniles and a low of 0% to a high of 17% in female juveniles. Bruising was the only finding in the adolescents and adult males. Erythema was observed only in juvenile animals, two males and three females. Generalized eyelid edema was observed only in juvenile animals, one male and one female. These animals had concurrent erythema in the affected eyelid. Animals with generalized eyelid edema and erythema are considered suspect for active tuberculosis and warrant further diagnostics. In this report, the most observed reaction among all age groups and sexes was bruising. Erythema and edema were rarely observed. Only juvenile animals were observed with either erythema, edema or both.

## Introduction

Non-human primates (NHP) imported to the United States must be quarantined per the CDC foreign animal quarantine regulations [1]. The regulations dictate specific requirements and testing necessary for NHPs to be approved for release to a regulated enterprise. An example of a requirement is the quarantine period must be a minimum 31 days, and an example of the diagnostic requirements is the animals must be tested negative for tuberculosis (*Tb*) using mammalian (human) old tuberculin (MOT) also known as Koch’s old tuberculin or old tuberculin for three consecutive tests completed at two-week intervals. The tuberculin skin test (TST) remains the gold standard screening method for *Tb* in NHPs, although other diagnostic modalities are available including testing serum by the multiplex fluorometric immune assay. Alternative tests are not recognized by the CDC for *Tb* screening.

Currently in laboratory animal medicine there is no published data or guidance regarding the quantity of reactions expected when testing NHPs with MOT. Therefore, we documented all reactions from 933 Mauritian origin *Macaca fascicularis* (cynomolgus macaque, long-tailed macaque) that were quarantined in 2017. The goals of this report are twofold. The first, to report the quantity and type of responses observed from all TST injections across the 933 animals. The second goal, to provide the laboratory animal medicine practitioner with reference ranges of reactions to MOT in Mauritian juvenile cynomolgus macaques.

## Methods

The primates in this report were *M*. *fascicularis* sourced from one supplier in Mauritius. The animals varied in sex, age, size and body weight. The animals were chosen by the supplier to meet selected health, sex, and age specifications set by the end user, not the quarantine facility. All animals had been tested a minimum of three times for *Tb* prior to export, using mammalian (bovine) purified protein derivative tuberculin (PPD) and at least one TST using MOT. Animals received multiple physical exams, were vaccinated minimally for measles, were tested negative for various NHP viral diseases such as Macacine *alphaherpesvirus* 1 (aka Herpes B virus or B virus) and simian immunodeficiency virus and receive various antiparasitic and anthelmintic treatment before reaching the US. Animal groups imported to our facility were of varying number, typically with a maximum of 112.

The practices and procedures performed in CDC quarantine in the US are solely veterinary in nature, akin to clinical veterinary practice. Therefore, there was no ethics committee approval required or requested to complete or perform diagnostic testing on the animals. Once the cohort has been judged through examination and testing by a veterinarian and fulfilling all requirements does the quarantine facility request release of the group from CDC. Once release is approved, then the animals may be transported to other facilities within the US. Animals can be transported to United States Department of Agriculture (USDA) licensed facilities. If animals are sent to a licensed research facility then they must be held under an ethics committee approved protocol. The animals held in our facility had no research or experimentation conducted. All veterinary care was completed and overseen by experienced veterinarians.

Upon arrival at our facility, animals were uncrated and housed in designated quarantine rooms. Animals were quartered in stainless steel wire cages sized according to body weight. Juvenile animals were paired at the start of the quarantine period and only separated in cases of incompatibility. Adolescent animals were paired and separated similarly to the juvenile animals. Both juvenile and adolescent animals were provided a new partner if a compatible animal was available. Adult male animals were singly housed unless specifically shipped with a compatible partner. There were no adult females quarantined in 2017. The rooms were ventilated with 100% fresh air, with greater than 10 air changes per hour. Room temperatures were maintained between 64–84°F, primarily between 73–78°F. The quarantine rooms were not air conditioned. Animals were fed a commercially prepared biscuit daily based on 3–4% of their body weight, along with a section of fresh fruit or vegetable daily. Animals were provided a measured amount of fresh water daily via PVC water tube with lixit as well as in a 4” or 6” diameter round PVC bowl. The animals were housed indoors with a 12 hour on-off light cycle. All animals were observed twice daily by trained and experienced staff for signs of illness or behavioral changes. An experienced veterinarian was always available to address any animal medical issues. All animals were provided a food treat three times per week, typically popcorn made by the facility. Each animal was provided a manipulable toy within the cage. Any animal housed singly, found with alopecia or a behavioral issue was provided additional enrichment consisting of such items as seeds within a small paper bag or a paper cup or discrete pieces of paper to shred. The facility is licensed by the USDA, CDC and is accredited by AAALAC.

All animals were anesthetized with ketamine dosed at approximately 5–8 mg/kg intramuscularly prior to handling, with additional ketamine administered as needed. All animals were weighed and given a physical examination as well as administered MOT. The TST procedure has previously been described [2]. 0.1 mL. of MOT was administered in an alternating upper eyelid per each TST set using a 27-gauge ½” needle attached to a 1 mL. tuberculin syringe. Two animals on one occasion each were not injected in the appropriate eyelid because of preexisting bruising. In these two cases MOT was injected in a documented and marked location on the ventral sternum for ease of visualization. Reading the TST requires visual observation of the injected eyelid or alternate skin site. In cases where there is uncertainty of a reaction at the 72-hour reading the animal is sedated with ketamine as described above and a veterinarian assesses the reaction. Reactions are documented then correlated to a previously published scoring system based on a scale of 0–5 [3]. The grading system is described as follows grade 0 (no reaction), grade 1 (bruising only), grade 2 (erythema only); both are considered negative reactions. Grade 3 (erythema and slight edema of entire eyelid) reactions are considered suspicious for *Tb*. Grade 4 (erythema with greater edema of entire eyelid with eyelid nearing full closure) and grade 5 (erythema with edema closing the eyelid and necrosis at the injection site) are considered positive reactions and a probable case of *Tb*. Although this is the generally accepted grading system it is not always 100% accurate. Depending on the quantity and grades of reactions of the cohort and other animal health factors including illness or death may increase the index of suspicion of *Tb* in the group [4]. In a case where skin other than the eyelid is injected with MOT, a visible wheal is evidence of a suspect or positive reaction.

All sets of TST were performed at 14-day intervals with two exceptions. The exceptions occurred when the second TST of two separate cohorts was administered 7 days after the first. This occurred because the first TST for each cohort had an animal with a grade 3 reaction. Facility procedure requires the next TST to be completed 7 days after a suspect or positive reactor is identified. All MOT was purchased from the same source and eight cohorts were tested using the same lot, the final cohort received a different lot. After TST injection, facility procedure requires the same trained and experienced person using a flashlight to assess the reactions at 24, 48 and 72 hours. When the animals are observed for a reaction at each time point, the assessor documents the observation. Subsequently the reaction is tracked and documented through the 72-hour final observation. The 72-hour observation is the determining assessment.

## Results

Nine cohorts of varying size, age and sex were quarantined in 2017. The nine cohorts totaled 933 animals. 933 animals entered quarantine with 929 surviving to completion. The 933 was composed of 490 males and 443 females. Of the 490 males starting quarantine, 488 completed their quarantine. 443 females started quarantine and 441 completed their quarantine. 52.5% of all animals were male with females comprising the remaining 47.5%. Animals were sorted between three age brackets for the purposes of this report. The first were juveniles ranging in age from one and one-half to three years old, next were adolescents aged from three up to eight years old and finally adults aged eight years and older. The population of this report is 933, of which 848 or 90.9% were juveniles. Juveniles were nearly split evenly by sex with 425 males and 423 females. Adolescents totaled 46 equaling 4.9% of the population. Adolescents were also nearly split evenly by sex with 21 males and 25 females. The remaining 4.2% of animals were 39 adults comprised solely of males. See Table 1.

**Table 1:** Total animal population by group, age and sex.

		All Ages		Juvenile		Adolescent		Adult	
Group #	Total Animal Number	M	F	M	F	M	F	M	F
1	100	50	50	50	50	0	0	0	0
2	112	56	56	56	56	0	0	0	0
3	112	56	56	56	56	0	0	0	0
4	112	56	56	56	56	0	0	0	0
5	97	36	61	36	36	0	25	0	0
6	89	62	27	29	27	10	0	23	0
7	107	62	45	55	45	5	0	2	0
8	106	56	50	50	50	5	0	1	0
9	98	56	42	42	42	1	0	13	0
Total	933	490	443	430	418	21	25	39	0

Seven of the nine cohorts equaling 720 animals received three TSTs per animal. All these animals survived to be released from quarantine. The two cohorts requiring additional TSTs equaled 213 animals. These animals received a total of six injections per animal, except for two animals from each cohort that received only one TST per animal. The remaining 209 animals survived to be released from quarantine. These two cohorts received additional TSTs as a result of one animal from each cohort presenting with a grade 3 reaction (slight edema and erythema in the injected eyelid at the 72-hour observation). Generalized slight eyelid edema represents a suspect *Tb* case (aka responder), and the decision was made to euthanize and necropsy these animals. The animals were euthanized with an overdose of a commercially prepared pentobarbital euthanasia solution after sedation with ketamine. After euthanasia the animals were necropsied and had the hilar lymph nodes, lung and other tissue submitted for acid fast staining, and culture. Test results demonstrated no evidence of *Tb*. Facility policy requires the cagemate of an animal euthanized with a suspicious or positive reaction also be euthanized. Per CDC regulation, once a suspicious or positive animal is removed from the group the remaining animals must undergo a minimum of five consecutive negative TSTs to be released from quarantine.

A total of 33 sets of TSTs were administered to all nine cohorts. A successful quarantine consists of a minimum of three consecutive negative TSTs with no clinical signs of disease. Seven cohorts received the minimum three TSTs each for a total of 21 sets. Two cohorts each with an animal with a grade 3 reaction received a total of six TSTs each for a total of 12 sets. One cohort had an animal with bruising, erythema and edema to the upper eyelid, that was judged by a veterinarian as focal edema to the eyelid and then graded 1–2. This group received no additional TSTs. These combined to equal 33 TST sets, resulting in 3418 individual TSTs being administered. 3124 or 91.4% of all injections were administered to juvenile animals. 1595 or 46.6% injections were administered to male juveniles and 1529 or 44.7% in female juveniles. Adolescent animals received a total of 198 injections, males received 93 and females 105. 80 injections were administered to the adult male animals.

Assessments made at the 72-hour time point are the officially documented test result for each animal. An animal may exhibit bruising, erythema and or edema at any time during the 72-hour evaluation period, but only the 72-hour observation is considered as the final determining assessment. It is only the 72-hour assessments that are reported here. Reactions to the TST were classified according to the observed clinical signs; these were bruising, erythema and edema. A total of 172 reactions were observed across all TSTs for all age groups and sexes. 164 or 95.3% of these reactions were bruises. Five or 2.9% of the reactions were classified as erythema. Three or 1.7% of the reactions were classified as edema. Juvenile animals had 94.2% or 162 of all reactions observed. Adolescents had 7 or 4.1% of the observed reactions and adult males 3 or 1.7% of all reactions. Male animals were observed to have 107 or 62.2% of all reactions, females had 65 or 37.8% of all reactions. All reactions by age category and sex are found in Table 2.

**Table 2:** The quantity and percent of TST reactions by age and sex.

	Age	Juvenile				Adolescent				Adult			
	Sex	M*	%	F*	%	M*	%	F*	%	M*	%	F	%
**Reaction**	**Bruise**	**96/172**	**55.8**	**58/172**	**33.7**	**4/172**	**2.3**	**3/172**	**1.7**	**3/172**	**1.7**	**NA**	**NA**
	**Erythema**	**2/172**	**1.2**	**3/172**	**1.7**	**0/172**	**0**	**0/172**	**0**	**0/172**	**0**	**NA**	**NA**
	**Edema**	**2/172**	**1.2**	**1/172**	**0.6**	**0/172**	**0**	**0/172**	**0**	**0/172**	**0**	**NA**	**NA**

*Ratio comprised of the numerator representing number of animals with a reaction of that age and sex and the denominator representing the total number of reactions observed.

Bruising was the most common reaction in all age groups and both sexes from all TST sets. Comparing reactions by animal age and sex from each TST set revealed bruising in juvenile males ranged from a low of 0% to a high of 30.2% and in juvenile females, ranging from a low of 0% to a high of 16.7%. Adolescent male bruising ranged from a low of 0% to a high of 40%. Adolescent female bruising ranged from a low of 0% to a high of 8%. Adult male bruising ranged from a low of 0% to a high of 7.7%.

The second most common reaction observed was erythema. Four of the 33 sets of TSTs had an animal(s) with erythema. Only juvenile animals in this report had observable erythema. Five juveniles had erythema that included two males and three females. Two male and one female from separate groups also had concurrent edema as previously described. Erythema ranged from a low of 0% to a high of 2.8% in male juveniles and in female juveniles ranged from a low of 0% to a high of 3.6% within a TST set.

The third and least common reaction observed was generalized edema of the injected eyelid. Across all 33 TST sets only two male juveniles and one female juvenile animal from separate cohorts had edema. Additionally, these animals also had erythema. As previously noted, one male was reclassified as a grade 1–2 after sedation and close inspection. See all reaction results from all TSTs in Table 3.

**Table 3:** All reactions observed at 72 hours after TST by group, age, and sex.

		TST Reactions												
		Bruise				Erythema				Edema				
Group	TST	M^#^	%	F^#^	%	M^#^	%	F^#^	%	M^#^	%	F	%	Age
1	1	0	0.0	0	0.0	0	0.0	0	0.0	0	0.0	0	0.0	Juvenile
	2	11/50	22.0	7/50	14.0	0	0.0	0	0.0	0	0.0	0	0.0	Juvenile
	3	1/50	2.0	0/50	0.0	0	0.0	0	0.0	0	0.0	0	0.0	Juvenile
2	1	1/56	1.8	5/56	8.9	0	0.0	0	0.0	0	0.0	0	0.0	Juvenile
	2	2/56	3.6	0	0.0	0	0.0	0	0.0	0	0.0	0	0.0	Juvenile
	3	0	0.0	0	0.0	0	0.0	0	0.0	0	0.0	0	0.0	Juvenile
3	1	0	0.0	0	0.0	0	0.0	0	0.0	0	0.0	0	0.0	Juvenile
	2	7/56	12.5	5/56	8.9	0	0.0	2/56	3.6	0	0.0	0	0.0	Juvenile
	3	1/56	1.8	5/56	8.9	0	0.0	0	0.0	0	0.0	0	0.0	Juvenile
4	1	0	0.0	1/56	1.8	0	0.0	0	0.0	0	0.0	0	0.0	Juvenile
	2	0	0.0	0	0.0	0	0.0	0	0.0	0	0.0	0	0.0	Juvenile
	3	0	0.0	1/56	1.8	0	0.0	0	0.0	0	0.0	0	0.0	Juvenile
5	1	9/36	25.0	1/36	2.8	1/36^a^	2.8	0	0.0	1/36^a^	2.8	0	0.0	Juvenile
		0	0.0	2/25	8.0	0	0.0	0	0.0	0	0.0	0	0.0	Adolescent
	2	1/36	2.8	0	0.0	0	0.0	0	0.0	0	0.0	0	0.0	Juvenile
	3	0	0.0	1/36	2.8	0	0.0	0	0.0	0	0.0	0	0.0	Adolescent
6	1	4/29	14.0	4/27	14.8	0	0.0	0	0.0	0	0.0	0	0.0	Juvenile
		1/10	10.0	0	0.0	0	0.0	0	0.0	0	0.0	0	0.0	Adolescent
		1/23	4.3	0	0.0	0	0.0	0	0.0	0	0.0	0	0.0	Adult
	2	0	0.0	0	0.0	0	0.0	0	0.0	0	0.0	0	0.0	Juvenile
	3	2/29	6.9	1/27	3.7	0	0.0	0	0.0	0	0.0	0	0.0	Juvenile
7	1	0	0.0	2/48	4.4	1/48^b^	2.1	0	0.0	1/48^b^	2.1	0	0.0	Juvenile
	2*	13/43^b^	30.2	8/48	16.7	0	0.0	0	0.0	0	0.0	0	0.0	Juvenile
		2/5	40.0	0	0.0	0	0.0	0	0.0	0	0.0	0	0.0	Adolescent
	3	0	0.0	0	0.0	0	0.0	0	0.0	0	0.0	0	0.0	Juvenile
	4	10/43	23.2	3/48	6.3	0	0.0	0	0.0	0	0.0	0	0.0	Juvenile
	5	5/43	11.6	2/48	4.2	0	0.0	0	0.0	0	0.0	0	0.0	Juvenile
	6	1/43	2.3	0	0.0	0	0.0	0	0.0	0	0.0	0	0.0	Juvenile
8	1	4/50	8.0	4/50	8.0	0	0.0	1/50^c^	2.0	0	0.0	1/50^c^	2.0	Juvenile
	2*	4/50	8.0	0	0.0	0	0.0	0	0.0	0	0.0	0	0.0	Juvenile
	3	2/50	4.0	1/48	2.1	0	0.0	0	0.0	0	0.0	0	0.0	Juvenile
	4	13/50	26.0	5/48	10.4	0	0.0	0	0.0	0	0.0	0	0.0	Juvenile
		1/5	20.0	0	0.0	0	0.0	0	0.0	0	0.0	0	0.0	Adolescent
	5	0	0.0	0	0.0	0	0.0	0	0.0	0	0.0	0	0.0	Juvenile
	6	0	0.0	1/48	2.1	0	0.0	0	0.0	0	0.0	0	0.0	Juvenile
9	1	3/42	7.1	0	0.0	0	0.0	0	0.0	0	0.0	0	0.0	Juvenile
		1/13	7.7	0	0.0	0	0.0	0	0.0	0	0.0	0	0.0	Adult
	2	1/42	2.4	1/42	2.4	0	0.0	0	0.0	0	0.0	0	0.0	Juvenile
	3	1/42	2.4	1/42	2.4	0	0.0	0	0.0	0	0.0	0	0.0	Juvenile
		1/13	7.7	0	0.0	0	0.0	0	0.0	0	0.0	0	0.0	Adult

^#^. Ratio comprised of the numerator representing number of animals with a reaction of that age and sex and the denominator representing the total number of animals of the cohort.

^a^. One male juvenile had a bruise, erythema and focal edema on the upper eyelid. This case was assessed under sedation as grade 1–2.

^b^. One male juvenile had both erythema and edema, a grade 3 reaction. Considered a suspect case of *Tb*. This animal and the cagemate were euthanized.

^c^. One female juvenile had both erythema and edema, a grade 3 reaction. Considered a suspect case of *Tb*. This animal and the cagemate were euthanized.

*. These two TSTs were completed after one week because of suspect *Tb* cases described above.

All TST injections were completed by the authors. Injector A completed three sets and injector B completed twenty-nine sets of TSTs. TST number twenty-six was completed by both authors. All reactions reported here at the 72-hour time point were observed by injector B, except for the twenty-sixth TST set which was completed by both authors. See results in Table 4.

**Table 4:** Comparison of all animal reactions to MOT at 72 hours by injector.

Injector	A			B		
TST	Bruise	Erythema	Edema	Bruise	Erythema	Edema
1	NA	NA	NA	0	0	0
2	NA	NA	NA	18	0	0
3	NA	NA	NA	1	0	0
4	NA	NA	NA	6	0	0
5	NA	NA	NA	2	0	0
6	0	0	0	NA	NA	NA
7	0	0	0	NA	NA	NA
8	NA	NA	NA	12	2	0
9	NA	NA	NA	6	0	0
10	NA	NA	NA	1	0	0
11	NA	NA	NA	0	0	0
12	NA	NA	NA	1	0	0
13	NA	NA	NA	12	1	1
14	NA	NA	NA	1	0	0
15	NA	NA	NA	1	0	0
16	NA	NA	NA	10	0	0
17	NA	NA	NA	0	0	0
18	NA	NA	NA	3	0	0
19	NA	NA	NA	2	1	1
20	NA	NA	NA	23	0	0
21	0	0	0	NA	NA	NA
22	NA	NA	NA	13	0	0
23	NA	NA	NA	7	0	0
24	NA	NA	NA	1	0	0
25	NA	NA	NA	8	1	1
26*	NA	NA	NA	4	0	0
27	NA	NA	NA	3	0	0
28	NA	NA	NA	19	0	0
29	NA	NA	NA	0	0	0
30	NA	NA	NA	1	0	0
31	NA	NA	NA	4	0	0
32	NA	NA	NA	2	0	0
33	NA	NA	NA	3	0	0

*This set of TST injections was completed by both authors.

All assessments were completed by both authors. Observer A (same designation as injector A) completed 18 assessments and observer B (same designation as injector B) completed 15. Observer A had a total of 19 recorded bruises versus 145 for observer B. Observer A recorded no animals with either erythema or edema. Observer B recorded all cases of erythema and edema. Individual animal reactions cannot be ascribed to a particular person for the 26^th^ TST set because the records reflect both authors as injectors. See Table 5 for the results.

**Table 5:** Comparison of all animal reactions to MOT at 72 hours by observer.

	A			B		
TST	Bruise	Erythema	Edema	Bruise	Erythema	Edema
1	0	0	0	NA	NA	NA
2	NA	NA	NA	18	0	0
3	1	0	0	NA	NA	NA
4	NA	NA	NA	6	0	0
5	NA	NA	NA	2	0	0
6	0	0	0	NA	NA	NA
7	0	0	0	NA	NA	NA
8	NA	NA	NA	12	2	0
9	NA	NA	NA	6	0	0
10	1	0	0	NA	NA	NA
11	0	0	0	NA	NA	NA
12	1	0	0	NA	NA	NA
13	NA	NA	NA	12	1	1
14	1	0	0	NA	NA	NA
15	1	0	0	NA	NA	NA
16	NA	NA	NA	10	0	0
17	0	0	0	NA	NA	NA
18	3	0	0	NA	NA	NA
19	NA	NA	NA	2	1	1
20	NA	NA	NA	23	0	0
21	0	0	0	NA	NA	NA
22	NA	NA	NA	13	0	0
23	NA	NA	NA	7	0	0
24	1	0	0	NA	NA	NA
25	NA	NA	NA	8	1	1
26	4	0	0	NA	NA	NA
27	3	0	0	NA	NA	NA
28	NA	NA	NA	19	0	0
29	0	0	0	NA	NA	NA
30	1	0	0	NA	NA	NA
31	NA	NA	NA	4	0	0
32	2	0	0	NA	NA	NA
33	NA	NA	NA	3	0	0
Sum	19	0	0	145	5	3

## Discussion

The current standard screening test for tuberculosis during quarantine is the TST utilizing MOT, as required by the CDC. This is a well-established diagnostic tool in NHPs that has been described and reported in the literature, including TST positive cases (grade 3–5) of laboratory confirmed tuberculosis. What has yet to be reported are the quantity and quality of reactions to the TST using MOT in a diverse population of NHPs, especially cynomolgus macaques. Here we report the quantity and quality of these reactions in a population of cynomolgus macaques held at our facility during 2017. In recent years the use of Mauritius origin *Macaca fascicularis* has significantly increased. Therefore, the publication of this information is relevant to the quarantine of these animals in the US and to the laboratory animal veterinarians that oversee their medical care. In addition to reporting our observations, we present ranges of reactions in juvenile cynomolgus macaques and considerations when comparing these reactions with other cynomolgus macaque groups.

The nine quarantine cohorts of this report resulted in a total of 33 TST sets administered. Juvenile animals received 91.4% (3124/3418) of all injections. Based on juveniles receiving most injections, it could be expected that juveniles represent the majority of reactions. This is the case, with juveniles having 94.2% (162/172) of all observed reactions. Adolescents and adults, excluding adult females, also had reactions, but these were limited to only bruising and in minor percentages. Other reactions, erythema and edema did occur, but only in juvenile animals. The quantity of these reactions consisted of minor percentages, three and two percent respectively.

When comparing all observed reactions, animal subpopulations and sexes there are some interesting similarities and differences. Juveniles represent 90.9% (848/933) of the total animal population and have 94.2% (162/172) of all reactions. Adolescents represent 4.9% (46/933) of the population and had 4.1% (7/172) of all reactions. Adult males were 4.2% (39/933) of the population and had only 1.7% (3/172) of all reactions. Juveniles and adolescents have similar proportions of reactions to their respective population numbers. Adult males have 50% fewer reactions as compared to the juveniles and adolescents. Males represented 52.5% (490/933) of the total population and females 47.5% (443/933). Males had 62.2% (107/172) of all reactions and females 37.8% (65/172). Males were nearly twice as likely to have had any reaction as compared to females, with the exception of one male and female juvenile with a grade 3 reaction. There appears to be an association between males and an increased likelihood of any reaction, especially bruising at the 72-hour assessment, with an equal chance of a grade 3 reaction in male and female juveniles.

Juvenile animals exhibited 89.5% of all bruises (154/164). This is comparable with juveniles representing 90.9% (848/933) of all animals in this report. Male juveniles had 62.3% (96/154) of observed bruising versus 37.7% (58/154) for females. Male juveniles are 46.1% (430/933) of the study population and females 44.9% (418/933). Male and female juveniles have comparable group size, with male juveniles over represented with eyelid bruising at 72 hours versus females. The quantity of female juvenile bruising is similar to their portion of the population although male juveniles had twice the number of bruises. The fact that males are nearly two times as likely to have been observed with eyelid bruising may be explained by several possibilities. Males may be predisposed to eyelid bruising due to hormonal, anatomic or physiologic differences than females, and males may have increased sensitivity to the chemical components of the tuberculin. The non-*Mycobacterial* chemical components of MOT are considerable [5]. When performing the TST, the needle must penetrate the epidermis and a pocket is created when the tuberculin is injected. This intradermal pocket leads to tissue trauma and bleeding. This is a probable source of some or all of the observed bruising. Less likelier causes of increased bruising in juvenile males include more aggressive injection technique and rougher tissue handling and bias in observer assessments between the sexes. These human factors although possible are not as likely as animal intrinsic factors or the injection itself since sex is not considered when performing the TST or observing for reactions. The proportion of adolescent male bruising is 2.4% (4/164) and for females it is 1.8% (3/164) of all bruises. Adolescents comprise 5% of this animal population, with males representing 2.3% and females 2.7%. The amount of bruising in male and female adolescents is in line with their percent of the total population. Possible explanations as to the cause of the bruising seen in adolescents is similar to that of the juvenile animals. Lastly, adult males represent 4.2% (39/933) of the population. Adult males experienced 3/164 bruises or 1.8% of all bruises. In comparison to the younger animals, adult males appear to have about half as much bruising versus their percent of the population. Continuing aging changes of the skin, hormones, physiology and a larger eyelid compared to younger animals may contribute to less trauma and less reactivity. Again, injector and observer bias are possible. But any bruising is probably due to direct tissue trauma from the injection or the MOT itself.

Bruising of the eyelid could be an expected sequela of an intradermal tuberculin injection since the TST requires deposition of the tuberculin between the layers of skin. In the eyelid there is no subcutaneous tissue so either the tuberculin is properly placed intradermally or there is a misdose. Confirmation of a properly placed intradermal injection is visualization of a small bleb at the site of injection. A properly placed intradermal injection forces the tuberculin to create a pocket between the epidermis and dermis, which can traumatize capillary and small blood vessels. Also, the direct trauma from the needle penetrating the epidermis and possibly the dermis can also account for vascular trauma and a resulting bruise. A misdose of tuberculin in the eyelid results in the needle penetrating completely through either the epidermis or dermis and if the injection is attempted no bleb will be observed. A misdose requires the animal be redosed with proper bleb visualization in the same eyelid if there is no observable bruise or an alternate location if the eyelid is bruised. Only in the case of an alternate site other than an eyelid such as the skin of the sternum or abdomen would subcutaneous dosing be possible. Lack of a bleb indicates a subcutaneous injection. An animal subcutaneously dosed ideally would not be redosed immediately, but rather a repeated proper intradermal injection should be performed seven days hence as the animal would receive twice as much antigen and may be more prone to have a false positive reaction.

Practitioners may expect 0–33% of juvenile males in a cohort to have bruising as a result of the TST with MOT, female juveniles 0–17%, adolescent males 0–40%, adolescent females 0–8% and adult males 0–8%. Bruising consistently at zero or above the stated ranges should be cause for concern. Cohorts with no animals observed with bruising necessitates a critique of injection technique, tuberculin storage and expiration and proper reaction assessment. Bruising reactions consistently above the stated percentages may be more difficult to evaluate. Bruising may be a natural consequence of intradermal injections or could be improper technique such as over handling of the skin, excessive movement of the needle after penetration of the epidermis, improper needle selection (26–27 needle gauge is recommended), or over interpreting reactions. It is clear that appropriately trained and experienced personnel making follow up visual assessments are a critical component to this process. In cases of an uncertain reaction, it is advised to sedate the animal at the 72-hour time point and have an experienced veterinarian evaluate the site. If an animal is sedated for evaluation, it is recommended to make a visual assessment first, then palpate the eyelid. Minor swelling can occur after digital palpation and cause false positive reactions. We palpate the control eyelid first then the injected eyelid, giving a good basis of comparison.

Bruising is not the only possible reaction to tuberculin. Animal age and possible previous infectious disease exposure may be factors in an animal’s reaction to tuberculin. Older animals have likely received more TSTs prior to arrival in the US (easily noted in the animal’s medical records, which are best reviewed prior to entry to the facility) due to longer duration in captivity. This may result in fewer cases of grade 3–5 reactions, since suspect and positive reactor animals should have been diagnosed and removed prior to shipment. The diligence and training of the veterinary staff at the point of origin are important quality control factors because the receiving quarantine facility is relying on their medical record documentation, skill in administering and assessing the TST, the availability of MOT which can be limited outside of the US, on the quality and storage of the tuberculin, and the facilities occupational health screening of all staff that come in to contact with the animals. This should include routine *Tb* testing and measles vaccination at a minimum.

The TST is our first line diagnostic tool to screen for active tuberculosis, but may not have the sensitivity required to screen for animals with latent tuberculosis. Also, some animals with *Tb* may be anergic and therefore unresponsive to MOT. Undiagnosed tuberculosis is always of concern to veterinarians. Facilities receiving animals should perform additional inhouse quarantine to screen for such animals and minimize the risk of spreading tuberculosis in a closed colony. The process of preparing an animal for intercontinental transport, including vaccination may suppress the response to tuberculin, which for example is the case when an animal is measles vaccinated within 28 days of a TST. This would eliminate or minimize an animal’s response to tuberculin causing a probable false negative reaction and if vaccinated the TST must be delayed until after the 28-day exclusion period. Stress during and after recent shipping is a well-known and documented phenomenon in animals. This stress response may suppress the immune system allowing a recrudesce of latent *Tb*. Reactivation of latent *Tb* may not be diagnosed with the TST until after an animal has cleared CDC quarantine.

Other possible reactions to MOT injection are erythema and edema. Erythema may occur due to similar causes as bruising or may be an immunologic reaction to the *Mtb* cellular fraction of the tuberculin. Erythema with concurrent edema in a patient increases the probability of a host immune response to the *Mtb* fraction of the MOT, a type IV hypersensitivity reaction. The greater the amount of edema closing the eyelid the greater probability of a true positive reaction. Increasing generalized eyelid edema drives the eyelid closer to closure and increases the thickness of the eyelid. Visually, in an unsedated animal, closing of the eyelid is most easily noted. Eyelid thickening is more difficult to assess with the animal conscious. Eyelid thickening is best appreciated with the animal sedated and in dorsal recumbency. In this position a direct comparison with the unaffected eye is easily made. Unless an animal has loss of the opposite eye or significant anatomic trauma to the opposite eyelid, this untreated eyelid is the basis for comparison to the treated eyelid. When the animal is sedated and after the visual exam, digital palpation of the eyelid can be performed and the increased thickness of the affected eyelid can be appreciated.

It is clear from the data that erythema is a rarely observed reaction. Five juvenile animals were observed with erythema; contrasted with no adolescents or adult males. Of the five animals, two were juvenile males and three were juvenile females. 85% or 28 of the 33 TST sets did not have an animal with erythema. Erythema ranged from a low of 0% to a high of 3% within a male juvenile cohort and females exhibited a low of 0% to a high of 4% within a female juvenile cohort. Therefore, the laboratory animal professional may expect erythema to be observed rarely in Mauritian cynomolgus macaques. If observed, it is most likely to occur in juvenile animals.

The rarest reaction observed was edema. Uniform eyelid edema occurred in only two juvenile animals, one male and one female. This reaction occurred in two different cohorts and two different TST sets. One male juvenile in one TST set had edema, consequently the remaining 32 TST sets had no male with edema. Males had a low of 0% to a high of 2.1% of animals in a cohort with edema. Alternatively, this can be quantified as 1/425 male juveniles had an observed grade 3 reaction. Female juveniles with observed edema (grade 3 reaction) ranged from a low of 0% in 32 of 33 TST sets to a high of 2% in one cohort. Similarly, this can be quantified as 1/423 female juveniles had a suspect *Tb* reaction. In NHPs, any edema observed at the 72-hour assessment is considered minimally a *Tb* suspect. Both animals reported here were necropsied and selected tissue submitted for culture and acid-fast staining. *Tb* was not identified or isolated, although not ruling out possible infection by *Tb*, it makes it much less likely. It is probable that these two animals had false positive reactions and had hypersensitivity reactions to the tuberculin or they could have been exposed or infected by nontuberculous mycobacteria. Animals exposed or infected with microorganisms such as *M*. *avium* or other non-tuberculous mycobacteria can have grade 3–5 reactions to MOT. The rate of a false positive reaction to MOT in cynomolgus macaques is not known.

Comparatively in the US, there exists a published performance standard when testing cattle for *M*. *bovis*. The USDA provides federally accredited veterinarians with a range of the expected number of suspect positive (aka responders) animals per a defined number of tested animals [6]. In cattle, bovine PPD tuberculin is injected intradermally in the caudal tail fold skin. The injection site must be visually inspected and palpated at 72 hours post injection by the same veterinarian. A suspect positive reaction is any thickening to the skin at the injection site. The standard states that for every 916 to 1050 animals the expected minimum number of responders is 5. This standard is a useful quality control for veterinarians to assess their performance when testing and making the injection site visualization and palpation. Too few responders may indicate such issues as improper injection technique and problems with tuberculin storage and handling. Too many responders may indicate active tuberculosis or cross reaction with other disease conditions such as Johne’s disease caused by *Mycobacterium avium* subspecies *paratuberculosis*. Either deviation warrants follow-up by the veterinarian.

Strengths of the data presented can be found in our TST reaction assessment procedure. We have the same trained and experienced personnel complete all three readings. This allows for greater consistency in observations and documentation. Animal reactions to tuberculin vary in intensity and duration. Utilizing the same personnel to make all observations allows consistent assessment of the waxing or waning of an individual animal’s response. Potential exists for laboratory animal professionals to vary in their observational skills. This may increase or decrease the quantity and quality of observed reactions skewing results. Determination of the type of reaction can be addressed at the final 72-hour reading with assessment by an experienced veterinarian. Observation of a TST reaction is a combination of science and art. Although there are three possible reactions to be observed, there is large variability in the behavior and anatomy of any animal as well as individual variation in tuberculin reaction intensity. Animal variables that increase the difficulty in completing accurate observation include continuous movement during observation, depth of the orbit and eyelid, intensity of eyelid skin pigmentation and rapidity of eye blinks. Room lighting can also be a factor in completing accurate assessments, as shadows in an animal cage are inevitable since most lighting indoors is projected from the ceiling, as is the case in this facility. Our TST observation procedure also includes using a focused light source when assessing reactions to create the best possible visibility of the eyelid. Should an animal have a suspicious, positive or difficult to assess reaction, then the animal should be sedated for reaction verification by a veterinarian. Atypical TST reactions and sedating of NHP for TST reading has been previously described [4].

A weakness to our data also resides in the assessment of the post injection reactions. This is evidenced by the large differential in animal reactions noted between observer A and B. Both are experienced and trained staff that have performed numerous TST injections, and assessed multiple thousands of NHPs for reaction to MOT. The observers have both experienced confirmed cases of tuberculosis (*Mtb*, *M*. *bovis*, *M*. *avium*) in NHP that were initially diagnosed with MOT. Additionally, the observers often discuss the reactions observed in a group and will in cases of an unclear reaction(s) perform a confirmatory visual assessment of the animal(s) in question. This cooperation allows additional depth of experience making observations and helps to guide further diagnostic steps including animal sedation.

In conclusion, bruising was the most common reaction observed, followed by erythema and then most rarely edema. Bruising (grade 1 reaction) in male juvenile cynomolgus macaques ranged from 0% to 33% and females from 0% to 24% in a cohort. Erythema (grade 2) in juvenile males ranged from 0% to 2.4% and females from 0% to 3.6% of a cohort. Lastly, edema with erythema (grade 3) in juveniles occurred in 1/425 males and 1/423 females of the population. Here we provide the laboratory animal practitioner with ranges of the number and type of reactions to be expected in juvenile Mauritian cynomolgus macaques administered MOT and ideas to use if their results are significantly different. In the future we hope that other institutions will publish their observational data to better inform the laboratory animal medicine community when performing TST assessments in NHPs.

## Supporting information

S1 Dataset Group 1. (PDF)

S2 Dataset Group 2. (PDF)

S3 Dataset Group 3. (PDF)

S4 Dataset Group 4. (PDF)

S5 Dataset Group 5. (PDF)

S6 Dataset Group 6. (PDF)

S7 Dataset Group 7. (PDF)

S8 Dataset Group 8. (PDF)

S9 Dataset Group 9. (PDF)
