# Supplementary material for: A retrospective analysis of the tuberculin skin test reactions of a single source population of Mauritian Macaca fascicularis held in quarantine during 2017
Source: PLoS One. 2022 Apr 14;17(4):e0265942. doi: 10.1371/journal.pone.0265942 (PMC9009605; doi:10.1371/journal.pone.0265942)
Supplement: S1 Dataset — (PDF) [file pone.0265942.s001.pdf]

TST Reaction Form

Room: C1 Group#: 01262017

Source: MU Species: Cx Total # animals in group: 100

			Date/Time/Initial 1/31/17 18:55			Date/Time/Initial 2/1/17 18:15			Date/Time/Initial 2/2/17 16:40		
			24 hr Reaction			48 hr Reaction			72 hr Reaction		
	Cage#	Animal#	Bruise	Red	Edema	Bruise	Red	Edema	Bruise	Red	Edema
1	4 (F)	[redacted]	B			<B			—		
2	11 (F)	[redacted]	<B			—			—		
3	19 (F)	[redacted]	<B			—			—		
4	20 (F)	[redacted]	<B			—			—		
5											
6			(4)								
7											
8											
9											
10											
11											
12											
13											
14											
15											
16											
17											
18											
19											
20											
		Total	4	0	0	1	0	0	0	0	0

Reaction Description		
B-bruise	R-red	E-edema
B-significant bruise	R-significant redness	E-significant edema
< B-diminishing bruise	<R-diminishing redness	<E-diminishing edema
B>-increasing bruise	R>-increasing redness	E>-increasing edema

TST Reaction Form

Room: C5 Group#: 01262017

Source: MU Species: Cy Total # animals in group: 100

	Cage#	Animal#	Date/Time/Initial 1/31/17 16:40 [initials] 24 hr Reaction			Date/Time/Initial 2/1/17 18:00 [initials] 48 hr Reaction			Date/Time/Initial 2/2/17 16:50 [initials] 72 hr Reaction		
			Bruise	Red	Edema	Bruise	Red	Edema	Bruise	Red	Edema
1	1 (M)	[redacted]	<B	[illegible]		—			—		
2	3 (M)	[redacted]	<B			—			—		
3	4 (M)	[redacted]	<B			<B			—		
4	12 (M)	[redacted]	<B			—			—		
5	15 (M)	[redacted]	<B			—			—		
6	25 (F)	[redacted]	B			—			—		
7	26 (F)	[redacted]	B			—			—		
8	23 (F)	[redacted]				<B			—		
9			(7)								
10											
11											
12											
13											
14											
15											
16											
17											
18											
19											
20											
		Total	7	0	0	2	0	0	0	0	0

Reaction Description		
B-bruise	R-red	E-edema
B-significant bruise	R-significant redness	E-significant edema
< B-diminishing bruise	<R-diminishing redness	<E-diminishing edema
B>-increasing bruise	R>-increasing redness	E>-increasing edema

TST Reaction Form

Room: CJ

Source: MU

Species: Cyno

Group#: 01262017

Total # animals in group: ~~64~~ 100 [illegible]

	Cage#	Animal#	Date/Time/Initial 14Feb17 1240 (MP)			Date/Time/Initial (MP) 15Feb17 [illegible]			Date/Time/Initial 16Feb17 (MP)		
			24 hr Reaction			48 hr Reaction			72 hr Reaction		
			Bruise	Red	Edema	Bruise	Red	Edema	Bruise	Red	Edema
1	1 (M)	[redacted]	<B			<B			—		
2	4 (M)	[redacted]	B			<B			<B		
3	5 (M)	[redacted]	<B			B			—		
4	(M)	[redacted]	B			<B			<B		
5	7 (M)	[redacted]	<B			—			—		
6	(M)	[redacted]	B			<B			<B		
7	11 (M)	[redacted]	B			<B			<B		
8	16 (M)	[redacted]	<B			<B			<B		
9	23 (F)	[redacted]	B			<B			<B		
10	24 (F)	[redacted]	<B			—			—		
11	27 (F)	[redacted]	<B			<B			—		
12	30 (M)	[redacted]	B			<B			<B		
13	32 (M)	[redacted]	>B			<B			<B		
14	33 (M)	[redacted]	B			<B			<B		
15	35 (M)	[redacted]	<B			<B			—		
16	36 (M)	[redacted]	B			<B			<B		
17	38 (M)	[redacted]	<B			<B			<B		
18											
19	8 (M)	[redacted]				<B			—		
20	40 (M)	[redacted]							<B		
		Total	17			16			12		
			14MJ 3FJ			15MJ 2FJ			11MJ 1FJ		

Reaction Description		
B-bruise	R-red	E-edema
B-significant bruise	R-significant redness	E-significant edema
< B-diminishing bruise	<R-diminishing redness	<E-diminishing edema
B>-increasing bruise	R>-increasing redness	E>-increasing edema

TST Reaction Form

Room: C1

Group#: 01262017

Source: MU

Species: Cymo

Total # animals in group: ~~36~~ 100

			Date/Time/Initial 14Feb17 1145A (MF)			Date/Time/Initial 15Feb17 (MF)			Date/Time/Initial 16Feb17 1145A (MF)		
			24 hr Reaction			48 hr Reaction			72 hr Reaction		
	Cage#	Animal#	Bruise	Red	Edema	Bruise	Red	Edema	Bruise	Red	Edema
1	4 (F)	[redacted]	B			<B			<B		
2	7 (F)		<B			<B			<B		
3	8 (F)		<B			<B			<B		
4	9 (F)		<B	[illegible]		<B			—		
5	11 (F)			<R		[illegible]	<R		<B		
6	19 (F)		<B			<B			~~<B~~		
7	(F)					<B			—		
8	18 (F)					<B			<B		
9	9 (F)					<B			—		
10	[illegible] (F)					<B			—		
11											
12			(5)	(1)		(9)	(1)		(6)		
13											
14											
15											
16											
17											
18											
19											
20											
		Total	5	1	0	9	1	0	6	0	0

5FJ 9FJ / 1FJ 6FJ

Reaction Description		
B-bruise	R-red	E-edema
B-significant bruise	R-significant redness	E-significant edema
< B-diminishing bruise	<R-diminishing redness	<E-diminishing edema
B>-increasing bruise	R>-increasing redness	E>-increasing edema

TST Reaction Form

Room: Cl Group#: 01262017

Source: MA Species: Cy Total # animals in group: 100

	Cage#	Animal#	Bruise	Red	Edema	Bruise	Red	Edema	Bruise	Red	Edema
			Date/Time/Initial 2/28/17 16:45			Date/Time/Initial 3/1/17 18:45 [illegible]			Date/Time/Initial 3/2/17 1330 [illegible]		
			24 hr Reaction			48 hr Reaction			72 hr Reaction		
1	3 (F)		B			—			—		
2	8 (F)	[redacted]	<B			—			—		
3	11 (F)	[redacted]	<B			—			—		
4	16 (F)	[redacted]	<B			—			—		
5											
6			(4)								
7											
8											
9											
10											
11											
12											
13											
14											
15											
16											
17											
18											
19											
20											
		Total	4	/	/	0	/	/	0	/	/

4(F)

Reaction Description		
B-bruise	R-red	E-edema
B-significant bruise	R-significant redness	E-significant edema
< B-diminishing bruise	<R-diminishing redness	<E-diminishing edema
B>-increasing bruise	R>-increasing redness	E>-increasing edema

TST Reaction Form

Room: C5

Group#: 01262017

Source: MU

Species: Cy

Total # animals in group: 100

			Date/Time/Initial 2/28/17 16:30 [illegible]			Date/Time/Initial 3/1/17 19:30 [illegible]			Date/Time/Initial 3/2/17 3:20 [illegible]		
			24 hr Reaction			48 hr Reaction			72 hr Reaction		
	Cage#	Animal#	Bruise	Red	Edema	Bruise	Red	Edema	Bruise	Red	Edema
1	10 (M)	[redacted]	<B			—			—		
2	13 (M)	[redacted]	B	<B		<B			<B		
3	15 (M)	[redacted]	<B			—			—		
4	16 (M)	[redacted]	B			—			—		
5											
6											
7											
8											
9											
10											
11											
12											
13											
14											
15											
16											
17											
18											
19											
20											
		Total	4	/	/	1	/	/	1	/	/

4MJ 1MJ 1MJ

Reaction Description		
B-bruise	R-red	E-edema
B-significant bruise	R-significant redness	E-significant edema
< B-diminishing bruise	<R-diminishing redness	<E-diminishing edema
B>-increasing bruise	R>-increasing redness	E>-increasing edema
