# Supplementary material for: A retrospective analysis of the tuberculin skin test reactions of a single source population of Mauritian Macaca fascicularis held in quarantine during 2017
Source: PLoS One. 2022 Apr 14;17(4):e0265942. doi: 10.1371/journal.pone.0265942 (PMC9009605; doi:10.1371/journal.pone.0265942)
Supplement: S2 Dataset — (PDF) [file pone.0265942.s002.pdf]

# TST Reaction Form

02232017

Group#: ~~02242017~~ am

Room: C2

Source: MN

Species: Cyno

Total # animals in group: 32 112

| | | | Date/Time/Initial 1Mar17 840A (MP) | | | Date/Time/Initial 2Mar17 222P (MP) | | | Date/Time/Initial 3Mar17 215P (MP) | | |
|---|---|---|---|---|---|---|---|---|---|---|---|
| | | | 24 hr Reaction | | | 48 hr Reaction | | | 72 hr Reaction | | |
| | Cage# | Animal# | Bruise | Red | Edema | Bruise | Red | Edema | Bruise | Red | Edema |
| 1 | 39 (M) | [illegible] | B | / | / | <B | / | / | <B | / | / |
| 2 | | | | | | | | | | | |
| 3 | | | | | | | | | | | |
| 4 | | | | | | | | | | | |
| 5 | | | | | | | | | | | |
| 6 | | | | | | | | | | | |
| 7 | | | | | | | | | | | |
| 8 | | | | | | | | | | | |
| 9 | | | | | | | | | | | |
| 10 | | | | | | | | | | | |
| 11 | | | | | | | | | | | |
| 12 | | | | | | | | | | | |
| 13 | | | | | | | | | | | |
| 14 | | | | | | | | | | | |
| 15 | | | | | | | | | | | |
| 16 | | | | | | | | | | | |
| 17 | | | | | | | | | | | |
| 18 | | | | | | | | | | | |
| 19 | | | | | | | | | | | |
| 20 | | | | | | | | | | | |
| | | Total | 1 | 0 | 0 | 1 | 0 | 0 | 1 | 0 | 0 |

| Reaction Description | | |
|---|---|---|
| B-bruise | R-red | E-edema |
| B-significant bruise | R-significant redness | E-significant edema |
| < B-diminishing bruise | <R-diminishing redness | <E-diminishing edema |
| B>-increasing bruise | R>-increasing redness | E>-increasing edema |

# TST Reaction Form

02232017

Room: C4

Source: MU

Species: Cyno

Group#: ~~02242017~~ [illegible]

Total # animals in group: 1/2

| | | | Date/Time/Initial 1Mar17 830A MP | | | Date/Time/Initial 2Mar17 232P MP | | | Date/Time/Initial 3Mar17 226P MP | | |
|---|---|---|---|---|---|---|---|---|---|---|---|
| | | | 24 hr Reaction | | | 48 hr Reaction | | | 72 hr Reaction | | |
| | Cage# | Animal# | Bruise | Red | Edema | Bruise | Red | Edema | Bruise | Red | Edema |
| 1 | 9 (F) | [redacted] | B | | | <B | | | <B | | |
| 2 | 23 (F) | [redacted] | B | | | <B | | | — | | |
| 3 | 24 (F) | [redacted] | B | | | <B | | | <B | | |
| 4 | 25 (F) | [redacted] | B | | | <B | | | — | | |
| 5 | 26 (F) | [redacted] | B | | | <B | | | — | | |
| 6 | 28 (F) | [redacted] | B | | | <B | | | <B | | |
| 7 | 29 (M) | [redacted] | B | | | <B | | | — | | |
| 8 | 35 (M) | [redacted] | / | | | <B | | | — | | |
| 9 | 19 (F) | [redacted] | / | | | <B | | | <B | | |
| 10 | (F) | [redacted] | / | | | B | | | <B | | |
| 11 | | | | | | | | | | | |
| 12 | | | | | | | | | | | |
| 13 | | | | | | | | | | | |
| 14 | | | | | | | | | | | |
| 15 | | | | | | | | | | | |
| 16 | | | | | | | | | | | |
| 17 | | | | | | | | | | | |
| 18 | | | | | | | | | | | |
| 19 | | | | | | | | | | | |
| 20 | | | | | | | | | | | |
| | | Total | 7 | / | / | 10 | / | / | 5 | / | / |

| Reaction Description | | |
|---|---|---|
| B-bruise | R-red | E-edema |
| B-significant bruise | R-significant redness | E-significant edema |
| < B-diminishing bruise | <R-diminishing redness | <E-diminishing edema |
| B>-increasing bruise | R>-increasing redness | E>-increasing edema |

31 Chest

# TST Reaction Form

Room: C2 Group#: 02232017

Source: MU Species: [illegible] Total # animals in group: 1/2

| | Cage# | Animal# | Date/Time/Initial 15Mar17 1152A [illegible] | | | Date/Time/Initial 16Mar17 [illegible] [illegible] | | | Date/Time/Initial 17Mar17 1055A [illegible] | | |
|---|---|---|---|---|---|---|---|---|---|---|---|
| | | | 24 hr Reaction | | | 48 hr Reaction | | | 72 hr Reaction | | |
| | | | Bruise | Red | Edema | Bruise | Red | Edema | Bruise | Red | Edema |
| 1 | 39 (M) | [redacted] | B | / | / | / | / | / | / | / | / |
| 2 | 40 (M) | [redacted] | B | / | / | / | / | / | / | / | / |
| 3 | | | | | | | | | | | |
| 4 | | | | | | | | | | | |
| 5 | | | | | | | | | | | |
| 6 | | | | | | | | | | | |
| 7 | | | | | | | | | | | |
| 8 | | | | | | | | | | | |
| 9 | | | | | | | | | | | |
| 10 | | | | | | | | | | | |
| 11 | | | | | | | | | | | |
| 12 | | | | | | | | | | | |
| 13 | | | | | | | | | | | |
| 14 | | | | | | | | | | | |
| 15 | | | | | | | | | | | |
| 16 | | | | | | | | | | | |
| 17 | | | | | | | | | | | |
| 18 | | | | | | | | | | | |
| 19 | | | | | | | | | | | |
| 20 | | | | | | | | | | | |
| | | Total | 2 | / | / | 0 | / | / | / | / | / |

| Reaction Description | | |
|---|---|---|
| B-bruise | R-red | E-edema |
| B-significant bruise | R-significant redness | E-significant edema |
| < B-diminishing bruise | <R-diminishing redness | <E-diminishing edema |
| B>-increasing bruise | R>-increasing redness | E>-increasing edema |

# TST Reaction Form

Room: C4

Source: MU

Species: Cyno

Group#: 02232017

Total # animals in group: 1/2

| | | | 24 hr Reaction | | | 48 hr Reaction | | | 72 hr Reaction | | |
|---|---|---|---|---|---|---|---|---|---|---|---|
| | | | Date/Time/Initial 15Mar17 1202P [illegible] | | | Date/Time/Initial 16Mar17 [illegible] | | | Date/Time/Initial 17Mar17 11A [illegible] | | |
| | Cage# | Animal# | Bruise | Red | Edema | Bruise | Red | Edema | Bruise | Red | Edema |
| 1 | 23 (F) | [redacted] | B | / | / | <B | / | / | / | / | / |
| 2 | 30 (M) | [redacted] | B | / | / | <B | / | / | — | — | — |
| 3 | 38 (M) | [redacted] | B | / | / | <B | / | / | / | B | B [illegible] |
| 4 | 32 (M) | [redacted] | / | / | / | <B | / | / | <B | / | / |
| 5 | 35 (M) | [redacted] | / | / | / | <B | / | / | <B | / | / |
| 6 | | | | | | | | | | | |
| 7 | | | | | | | | | | | |
| 8 | | | | | | | | | | | |
| 9 | | | | | | | | | | | |
| 10 | | | | | | | | | | | |
| 11 | | | | | | | | | | | |
| 12 | | | | | | | | | | | |
| 13 | | | | | | | | | | | |
| 14 | | | | | | | | | | | |
| 15 | | | | | | | | | | | |
| 16 | | | | | | | | | | | |
| 17 | | | | | | | | | | | |
| 18 | | | | | | | | | | | |
| 19 | | | | | | | | | | | |
| 20 | | | | | | | | | | | |
| | | Total | 3 | / | / | 5 | / | / | 2 | / | / |

| Reaction Description | | |
|---|---|---|
| B-bruise | R-red | E-edema |
| B-significant bruise | R-significant redness | E-significant edema |
| < B-diminishing bruise | <R-diminishing redness | <E-diminishing edema |
| B>-increasing bruise | R>-increasing redness | E>-increasing edema |

# TST Reaction Form

Room: C2 | Group#: 02232017

Source: [illegible] | Species: Cy | Total # animals in group: ~~106~~ 112 3/31/17 17:23 [illegible]

| | | | Date/Time/Initial 3/29/17 8:10 [illegible] | | | Date/Time/Initial 3/30/17 8:55 [illegible] | | | Date/Time/Initial 3/31/17 [illegible] | | |
|---|---|---|---|---|---|---|---|---|---|---|---|
| | | | 24 hr Reaction | | | 48 hr Reaction | | | 72 hr Reaction | | |
| | Cage# | Animal# | Bruise | Red | Edema | Bruise | Red | Edema | Bruise | Red | Edema |
| 1 | 15 (M) | [redacted] | <B | | | — | | | — | | |
| 2 | 31 (M) | [redacted] | B | | | — | | | — | | |
| 3 | 45 (M) | [redacted] | <B | | | — | | | — | | |
| 4 | | | | | | | | | | | |
| 5 | | | | | | | | | | | |
| 6 | | | | | | | | | | | |
| 7 | | | | | | | | | | | |
| 8 | | | | | | | | | | | |
| 9 | | | | | | | | | | | |
| 10 | | | | | | | | | | | |
| 11 | | | | | | | | | | | |
| 12 | | | | | | | | | | | |
| 13 | | | | | | | | | | | |
| 14 | | | | | | | | | | | |
| 15 | | | | | | | | | | | |
| 16 | | | | | | | | | | | |
| 17 | | | | | | | | | | | |
| 18 | | | | | | | | | | | |
| 19 | | | | | | | | | | | |
| 20 | | | | | | | | | | | |
| | | Total | 3 | Ø | Ø | Ø | Ø | Ø | Ø | Ø | Ø |

| Reaction Description | | |
|---|---|---|
| B-bruise | R-red | E-edema |
| B-significant bruise | R-significant redness | E-significant edema |
| < B-diminishing bruise | <R-diminishing redness | <E-diminishing edema |
| B>-increasing bruise | R>-increasing redness | E>-increasing edema |

# TST Reaction Form

Room: C4 Group#: 02232017

Source: MU Species: Cyno Total # animals in group: ~~106~~ 112

| | | | Date/Time/Initial 3/29/17 17:30 [initial] | | | Date/Time/Initial 3/30/17 18:40 [initial] | | | Date/Time/Initial 3/31/17 17:20 [initial] | | |
|---|---|---|---|---|---|---|---|---|---|---|---|
| | | | 24 hr Reaction | | | 48 hr Reaction | | | 72 hr Reaction | | |
| | Cage# | Animal# | Bruise | Red | Edema | Bruise | Red | Edema | Bruise | Red | Edema |
| 1 | 30 (M) | [illegible] | LB | | | — | | | — | | |
| 2 | | | | | | | | | | | |
| 3 | | | | | | | | | | | |
| 4 | | | | | | | | | | | |
| 5 | | | | | | | | | | | |
| 6 | | | | | | | | | | | |
| 7 | | | | | | | | | | | |
| 8 | | | | | | | | | | | |
| 9 | | | | | | | | | | | |
| 10 | | | | | | | | | | | |
| 11 | | | | | | | | | | | |
| 12 | | | | | | | | | | | |
| 13 | | | | | | | | | | | |
| 14 | | | | | | | | | | | |
| 15 | | | | | | | | | | | |
| 16 | | | | | | | | | | | |
| 17 | | | | | | | | | | | |
| 18 | | | | | | | | | | | |
| 19 | | | | | | | | | | | |
| 20 | | | | | | | | | | | |
| | | Total | 1 | Ø | Ø | Ø | Ø | Ø | Ø | Ø | Ø |

| Reaction Description | | |
|---|---|---|
| B-bruise | R-red | E-edema |
| B-significant bruise | R-significant redness | E-significant edema |
| < B-diminishing bruise | <R-diminishing redness | <E-diminishing edema |
| B>-increasing bruise | R>-increasing redness | E>-increasing edema |
