# Supplementary material for: A retrospective analysis of the tuberculin skin test reactions of a single source population of Mauritian Macaca fascicularis held in quarantine during 2017
Source: PLoS One. 2022 Apr 14;17(4):e0265942. doi: 10.1371/journal.pone.0265942 (PMC9009605; doi:10.1371/journal.pone.0265942)
Supplement: S3 Dataset — (PDF) [file pone.0265942.s003.pdf]

TST Reaction Form

Room: ~~C2~~ C1

Group#: 03302017

Source: Mu

Species: Cy

Total # animals in group: 112

			Date/Time/Initial 4/4/17 11:55 [illegible]			Date/Time/Initial 4/5/17 18:55 [illegible]			Date/Time/Initial 4/6/17 18:10 [illegible]		
			24 hr Reaction			48 hr Reaction			72 hr Reaction		
	Cage#	Animal#	Bruise	Red	Edema	Bruise	Red	Edema	Bruise	Red	Edema
1	8 (F)	[redacted]	B			<			—		
2	16 (F)	[redacted]	B			<			—		
3											
4											
5											
6											
7											
8											
9											
10											
11											
12											
13											
14											
15											
16											
17											
18											
19											
20											
		Total	2	Ø	Ø	2	Ø	Ø	Ø	Ø	Ø

Reaction Description		
B-bruise	R-red	E-edema
<u>B</u>-significant bruise	<u>R</u>-significant redness	<u>E</u>-significant edema
< B-diminishing bruise	<R-diminishing redness	<E-diminishing edema
B>-increasing bruise	R>-increasing redness	E>-increasing edema

TST Reaction Form

Room: C1

Source: NN

Species: cyno

Group#: 03302017

Total # animals in group: ~~36~~ 112

			Date/Time/Initial 2May17 747A [illegible]			Date/Time/Initial 3May17 920A [illegible]			Date/Time/Initial 4May17 8A [illegible]		
			24 hr Reaction			48 hr Reaction			72 hr Reaction		
	Cage#	Animal#	Bruise	Red	Edema	Bruise	Red	Edema	Bruise	Red	Edema
1	1 (F)	[redacted]	B	/	/	<B	/	/	<B	/	/
2	2 (F)	[redacted]	B	/	/	<B	/	/	<B	/	/
3	(F)	[redacted]	<B	/	/	<B	/	/	/	/	/
4	3 (F)	[redacted]	B	/	/	<B	/	/	<B	/	/
5	(F)	[redacted]	<B	/	/	/	/	/	/	/	/
6	17 (F)	[redacted]	<B	/	/	<B	/	/	/	/	/
7	5 (F)	[redacted]	—	—	—	<B	/	/	<B	/	/
8											
9											
10											
11											
12											
13											
14											
15											
16											
17											
18											
19											
20											
		Total	6	/	/	6	/	/	4	/	/

Reaction Description		
B-bruise	R-red	E-edema
B-significant bruise	R-significant redness	E-significant edema
< B-diminishing bruise	<R-diminishing redness	<E-diminishing edema
B>-increasing bruise	R>-increasing redness	E>-increasing edema

C1

TST Reaction Form

Room: ~~C7~~

Group#: 03302017

Source: MU

Species: Cynos

Total # animals in group: ~~36~~ 112

	Cage#	Animal#	Date/Time/Initial MP 18Apr17 1205P			Date/Time/Initial 19Apr17 1207pMP			Date/Time/Initial 20Apr17 2PM MP			
			24 hr Reaction			48 hr Reaction			72 hr Reaction			
			Bruise	Red	Edema	Bruise	Red	Edema	Bruise	Red	Edema	
1	2	F [redacted]	B			<B			<B			
2	3	F [redacted]	B			<B			<B			
3		F [redacted]	B			<B			—			
4	8	F [redacted]	B			<B			—			
5	9	F [redacted]	<B			<B			—			
6	10	F [redacted]	~~R~~	R		~~SR~~	<R			<R		SR.
7	18	F [redacted]	~~<B~~ ~~<B~~	<R		~~<B~~	<R			<R		<B
8		F [redacted]	<B			<B			<B			
9	15	F [redacted]				<B			<B			
10												
11												
12												
13												
14												
15												
16												
17												
18												
19												
20												
		Total	6	2	0	7	2	0	4	2	0	

9 277 Redness

Reaction Description		
B-bruise	R-red	E-edema
B-significant bruise	R-significant redness	E-significant edema
< B-diminishing bruise	<R-diminishing redness	<E-diminishing edema
B>-increasing bruise	R>-increasing redness	E>-increasing edema

TST Reaction Form

Room: C5

Source: MU

Species: Cyno

Group#: 03302017

Total # animals in group: ~~80~~ 11 2

			Date/Time/Initial 2May17 735A [illegible]			Date/Time/Initial 3May17 10A [illegible]			Date/Time/Initial 4May17 810A [illegible]		
			24 hr Reaction			48 hr Reaction			72 hr Reaction		
	Cage#	Animal#	Bruise	Red	Edema	Bruise	Red	Edema	Bruise	Red	Edema
1	16 (M)	[redacted]	<B	X	X	—	—	—	—	—	—
2	23 (M)	[redacted]	<B	X	X	<B	—	—	—	—	—
3	31 (F)	[redacted]	<B	X	X	—	—	—	—	—	—
4	(F)	[redacted]	<B	X	X	—	—	—	—	—	—
5	33 (M)	[redacted]	<B	X	X	—	—	—	—	—	—
6	34 (M)	[redacted]	<B	X	X	—	—	—	—	—	—
7	32 (F)	[redacted]	—	—	—	<B	—	—	<B	—	—
8	17 (M)	[redacted]	—	—	—	<B	—	—	—	—	—
9	7 (M)	[redacted]	—	—	—	<B	—	—	—	—	—
10	4 (M)	[redacted]	—	—	—	<B	—	—	/	/	/
11	16 (M)	—	—	—	—	—	—		<B	/	/
12											
13											
14											
15											
16											
17											
18											
19											
20											
		Total	6	/	/	5	/	/	2	/	/

Reaction Description		
B-bruise	R-red	E-edema
<u>B</u>-significant bruise	<u>R</u>-significant redness	<u>E</u>-significant edema
< B-diminishing bruise	<R-diminishing redness	<E-diminishing edema
B>-increasing bruise	R>-increasing redness	E>-increasing edema

C515.

TST Reaction Form

Room: C5 | Group#: 03302017

Source: MY | Species: CY | Total # animals in group: 112

	Cage#	Animal#	Date/Time/Initial 4/4/17 11:40 [illegible]			Date/Time/Initial 4/5/17 18:40 [illegible]			Date/Time/Initial 4/6/17 17:50 [illegible]		
			24 hr Reaction			48 hr Reaction			72 hr Reaction		
	Cage#	Animal#	Bruise	Red	Edema	Bruise	Red	Edema	Bruise	Red	Edema
1	5 (M)	[redacted]	<B			<<B			—		
2	16 (M)	[redacted]	<B			<B			—		
3	22 (M)	[redacted]	<B			<B			—		
4	38 (M)	[redacted]	B			<B			—		
5											
6											
7											
8											
9											
10											
11											
12											
13											
14											
15											
16											
17											
18											
19											
20											
		Total	4 5	Ø	Ø	4	Ø	Ø	Ø	Ø	Ø

Reaction Description		
B-bruise	R-red	E-edema
B-significant bruise	R-significant redness	E-significant edema
< B-diminishing bruise	<R-diminishing redness	<E-diminishing edema
B>-increasing bruise	R>-increasing redness	E>-increasing edema

TST Reaction Form

Room: C5 Group#: 03302017

Source: MU Species: Cyno Total # animals in group: ~~74~~ 112

	Cage#	Animal#	24 hr Reaction (Date/Time/Initial: 18Apr17 1214 MP)			48 hr Reaction (Date/Time/Initial: 19Apr17 1150 AMP)			72 hr Reaction (Date/Time/Initial)		
			Bruise	Red	Edema	Bruise	Red	Edema	Bruise	Red	Edema
1	1 (F)	[redacted]	<B			/			/		
2	2 (M)	[redacted]	<B			<B			<B		
3	11 (M)	[redacted]	<B			/			/		
4	(M)		>			<B			<B		
5	12 (M)	[redacted]	<B x2			<B			<B		
6	17 (M)	[redacted]	<B			<B			<B		
7	24 (M)	[redacted]	<B			<B			<B		
8	26 (F)	[redacted]	B			<B			<B		
9	27 (F)	[redacted]	<B			<B			—		
10	28 (F)	[redacted]	<B			<B			—		
11	36 (M)	[redacted]	<B			<B			—		
12	30 (M)	[redacted]				<B			<B		
13											
14											
15											
16											
17											
18											
19											
20											
		Total	12	0	0	11	0	0	7	0	0

Reaction Description		
B-bruise	R-red	E-edema
<u>B</u>-significant bruise	<u>R</u>-significant redness	<u>E</u>-significant edema
< B-diminishing bruise	<R-diminishing redness	<E-diminishing edema
B>-increasing bruise	R>-increasing redness	E>-increasing edema
