# Supplementary material for: A retrospective analysis of the tuberculin skin test reactions of a single source population of Mauritian Macaca fascicularis held in quarantine during 2017
Source: PLoS One. 2022 Apr 14;17(4):e0265942. doi: 10.1371/journal.pone.0265942 (PMC9009605; doi:10.1371/journal.pone.0265942)
Supplement: S4 Dataset — (PDF) [file pone.0265942.s004.pdf]

TST Reaction Form

Room: C2

Group#: 04072017

Source: Mu

Species: Cy

Total # animals in group: 112

	Cage#	Animal#	24 hr Reaction<br>Date/Time/Initial<br>5/9/17 15:15 [illegible]			48 hr Reaction<br>Date/Time/Initial<br>5/10/17 11:50 [illegible]			72 hr Reaction<br>Date/Time/Initial<br>5/11/17 17:25 [illegible]		
			Bruise	Red	Edema	Bruise	Red	Edema	Bruise	Red	Edema
1	31 (M)	[redacted]	<B			<<B			—		
2	32 (M)	[redacted]	<B			—			—		
3	33 (M)	[redacted]	B			<B			—		
4	36 (M)	[redacted]	B			<B			—		
5	44 (M)	[redacted]	B			<B			—		
6											
7											
8											
9											
10											
11											
12											
13											
14											
15											
16											
17											
18											
19											
20											
		Total	5	Ø	Ø	4	Ø	Ø	Ø	Ø	Ø

Reaction Description		
B-bruise	R-red	E-edema
B-significant bruise	R-significant redness	E-significant edema
< B-diminishing bruise	<R-diminishing redness	<E-diminishing edema
B>-increasing bruise	R>-increasing redness	E>-increasing edema

TST Reaction Form

Room: C 4 Group#: 04072017

Source: MY Species: Cy Total # animals in group: 112

	Cage#	Animal#	Date/Time/Initial 5/9/17 15:00 [initials]			Date/Time/Initial 5/10/17 11:30 [initials]			Date/Time/Initial 5/11/17 17:10 [initials]		
			24 hr Reaction			48 hr Reaction			72 hr Reaction		
			Bruise	Red	Edema	Bruise	Red	Edema	Bruise	Red	Edema
1	1 (M)	[illegible]	<B			<<B			—		
2	7 (F)	[illegible]	B			<<B			—		
3	23 (M)	[illegible]	<B			—			—		
4	24 (M)	[illegible]	<B			<<B			—		
5	28 (M)	[illegible]	B			<B			—		
6	29 (F)	[illegible]	B			<B			—		
7	9 (F)	[illegible]				B			(<B)		
8	15 (F)	[illegible]				<B			—		
9											
10											
11											
12											
13											
14											
15											
16											
17											
18											
19											
20											
		Total	6	0	0	~~7~~ 8 (initials) 10Oct18	~~0~~	0	1	0	0

Reaction Description		
B-bruise	R-red	E-edema
B-significant bruise	R-significant redness	E-significant edema
< B-diminishing bruise	<R-diminishing redness	<E-diminishing edema
B>-increasing bruise	R>-increasing redness	E>-increasing edema

TST Reaction Form

Room: C4

Group#: 04072017

Source: MU

Species: CY

Total # animals in group: 11 [illegible]

			Date/Time/Initial 4/25/17 18:45 [initials]			Date/Time/Initial 4/26/17 18:50 [initials]			Date/Time/Initial 4/27/17 19:00 [initials]		
			24 hr Reaction			48 hr Reaction			72 hr Reaction		
	Cage#	Animal#	Bruise	Red	Edema	Bruise	Red	Edema	Bruise	Red	Edema
1	18 (M)	[redacted]	<B			<<B			—		
2	22 (F)	[redacted]	<B			<B			—		
3	28 (M)	[redacted]	B			<B			—		
4	33 (M)	[redacted]	<B			<B			—		
5											
6											
7											
8											
9											
10											
11											
12											
13											
14											
15											
16											
17											
18											
19											
20											
		Total	4	0	0	4	0	0	0	0	0

Reaction Description		
B-bruise	R-red	E-edema
B-significant bruise	R-significant redness	E-significant edema
< B-diminishing bruise	<R-diminishing redness	<E-diminishing edema
B>-increasing bruise	R>-increasing redness	E>-increasing edema

TST Reaction Form

Room: C2

Group#: 04072017

Source: MU

Species: Cy

Total # animals in group: 112

			Date/Time/Initial 4/25/17 1750 [illegible]			Date/Time/Initial 4/26/17 18:40 [illegible]			Date/Time/Initial 4/27/17 [illegible] 18:10		
			24 hr Reaction			48 hr Reaction			72 hr Reaction		
	Cage#	Animal#	Bruise	Red	Edema	Bruise	Red	Edema	Bruise	Red	Edema
1	9	M [illegible]	<B			<B			–		
2											
3											
4											
5											
6											
7											
8											
9											
10											
11											
12											
13											
14											
15											
16											
17											
18											
19											
20											
		Total	1	0	0	1	Ø	Ø	Ø	Ø	Ø

Reaction Description		
B-bruise	R-red	E-edema
B-significant bruise	R-significant redness	E-significant edema
< B-diminishing bruise	<R-diminishing redness	<E-diminishing edema
B>-increasing bruise	R>-increasing redness	E>-increasing edema

TST Reaction Form

Room: C4 Group#: 04072017

Source: MY Species: Cy Total # animals in group: 112

			Date/Time/Initial 4/11/17 20:20 [illegible]			Date/Time/Initial 4/12/17 19:30 [illegible]			Date/Time/Initial 4/13/17 18:20 [illegible]		
			24 hr Reaction			48 hr Reaction			72 hr Reaction		
	Cage#	Animal#	Bruise	Red	Edema	Bruise	Red	Edema	Bruise	Red	Edema
1	1 (M)	[redacted]	<B	0		~			—		
2	7 (F)	[redacted]	<B			~			—		
3	11 (F)	[redacted]	<B			<B			<B		
4	26 (M)	[redacted]	<B			<B			—		
5	13 (F)	[redacted]	B			<B			—		
6											
7											
8											
9											
10											
11											
12											
13											
14											
15											
16											
17											
18											
19											
20											
		Total	~~5~~ 6	∅	∅	3	∅	∅	1	∅	∅

Reaction Description		
B-bruise	R-red	E-edema
B-significant bruise	R-significant redness	E-significant edema
< B-diminishing bruise	<R-diminishing redness	<E-diminishing edema
B>-increasing bruise	R>-increasing redness	E>-increasing edema

TST Reaction Form

Room: C2 Group#: 04072017

Source: MU Species: Cy Total # animals in group: 112

			Date/Time/Initial 4/11/17 20:40			Date/Time/Initial 4/12/17 19:40			Date/Time/Initial 4/13/17 18:30		
			24 hr Reaction			48 hr Reaction			72 hr Reaction		
	Cage#	Animal#	Bruise	Red	Edema	Bruise	Red	Edema	Bruise	Red	Edema
1	31 (M)	[redacted]	B			<B			—		
2	44 (M)	[redacted]	B			<B			—		
3											
4											
5											
6											
7											
8											
9											
10											
11											
12											
13											
14											
15											
16											
17											
18											
19											
20											
		Total	2	0	0	2	0	0	0	0	0

Reaction Description		
B-bruise	R-red	E-edema
B-significant bruise	R-significant redness	E-significant edema
< B-diminishing bruise	<R-diminishing redness	<E-diminishing edema
B>-increasing bruise	R>-increasing redness	E>-increasing edema
