# Supplementary material for: A retrospective analysis of the tuberculin skin test reactions of a single source population of Mauritian Macaca fascicularis held in quarantine during 2017
Source: PLoS One. 2022 Apr 14;17(4):e0265942. doi: 10.1371/journal.pone.0265942 (PMC9009605; doi:10.1371/journal.pone.0265942)
Supplement: S5 Dataset — (PDF) [file pone.0265942.s005.pdf]

TST Reaction Form

Room: C5 Source: MU Species: CY Group#: 05182017

Flashlight: (Yes) / No Total # animals in group: 97

			Date/Time/Initial 6/20/17 13:45 [illegible]			Date/Time/Initial 6/21/17 18:10 [illegible]			Date/Time/Initial 6/22/17 16:40 [illegible]		
			24 hr Reaction			48 hr Reaction			72 hr Reaction		
	Cage#	Animal#	Bruise	Red	Edema	Bruise	Red	Edema	Bruise	Red	Edema
1	10 (F)	[redacted]	<B	(J)		<B			—		
2	23 (M)	[redacted]	<B	(J)		—					
3	26 (M)	[redacted]	<B	(J)		—					
4	40 (M)	[redacted]	<B	(J)		—					
5											
6											
7											
8											
9											
10											
11											
12											
13											
14											
15											
16											
17											
18											
19											
20											
		Total	4	Ø	Ø	1	Ø	Ø	Ø	Ø	Ø

Reaction Description		
B-bruise	R-red	E-edema
B-significant bruise	R-significant redness	E-significant edema
< B-small/diminishing bruise	<R-slight/diminishing redness	<E-diminishing edema
B>-large/increasing bruise	R>-intense/increasing redness	E>-increasing edema

TST Reaction Form

Room: C3 Source: MU Species: CY Group#: 05182017

Flashlight: Yes / No (Yes circled) Total # animals in group: 97

			Date/Time/Initial 6/20/17 14:05 [illegible]			Date/Time/Initial 6/21/17 18:20 [illegible]			Date/Time/Initial 6/22/17 16:55 [illegible]		
			24 hr Reaction			48 hr Reaction			72 hr Reaction		
	Cage#	Animal#	Bruise	Red	Edema	Bruise	Red	Edema	Bruise	Red	Edema
1	6 (F)	[redacted]	B	Adol		<B			<B		
2	8 (F)	[redacted]	<B	Adol		<<B			—		
3	48 (F)	[redacted]	<B	(J)		<<B			—		
4	(M)										
5											
6											
7											
8											
9											
10											
11											
12											
13											
14											
15											
16											
17											
18											
19											
20											
		Total	3	Ø	Ø	3	Ø	Ø	1	Ø	Ø

Reaction Description		
B-bruise	R-red	E-edema
B-significant bruise	R-significant redness	E-significant edema
< B-small/diminishing bruise	<R-slight/diminishing redness	<E-diminishing edema
B>-large/increasing bruise	R>-intense/increasing redness	E>-increasing edema

TST Reaction Form

Room: C3 Source: MY Species: CY Group#: 05182017

Flashlight: Yes (circled) / No Total # animals in group: 97

	Cage#	Animal#	Date/Time/Initial 6/6/17 20:00 [illegible] 24 hr Reaction Bruise	Red	Edema	Date/Time/Initial 6/7/17 18:15 [illegible] 48 hr Reaction Bruise	Red	Edema	Date/Time/Initial 6/8/17 19:45 [illegible] 72 hr Reaction Bruise	Red	Edema
1	35 (M)	[redacted]	<B	(J.)		<<B			—	Ø	
2	38 (M)	[redacted]	<B	(J)		—			—		
3	44 (M)	[redacted]	B	(J.)		<B			—		
4	46 (M)	[redacted]	<B	(J.)		—			—		
5											
6											
7											
8											
9											
10											
11											
12											
13											
14											
15											
16											
17											
18											
19											
20											
		Total	4	Ø	Ø	2	Ø	Ø	Ø	Ø	Ø

Reaction Description		
B-bruise	R-red	E-edema
B-significant bruise	R-significant redness	E-significant edema
< B-small/diminishing bruise	<R-slight/diminishing redness	<E-diminishing edema
B>-large/increasing bruise	R>-intense/increasing redness	E>-increasing edema

TST Reaction Form

Room: CS Source: MU Species: Cp Group#: 05182017

Flashlight: (Yes) / No Total # animals in group: 97

			Date/Time/Initial 6/6/17 19:30 [illegible]			Date/Time/Initial 6/7/17 8:00 [illegible]			Date/Time/Initial 6/8/17 19:20 [illegible]		
			24 hr Reaction			48 hr Reaction			72 hr Reaction		
	Cage#	Animal# ✓	Bruise	Red	Edema	Bruise	Red	Edema	Bruise	Red	Edema
1	17 (F)	[redacted]	B	(J)		<B			<		
2	27 (M)	[redacted]	B	(J)		<B			—		
3	28 (M)	[redacted]	B	(J)		<B			<		
4	31 (M)	[redacted]	<B	(J)		<<B			<		
5	38 (M)	[redacted]	B	(J)		B			<B		
6	8 (F)	[redacted]	B	(J)		<B			—		
7		[redacted]									
8											
9											
10											
11											
12											
13											
14	17	[redacted] (J) (J)									
15											
16											
17											
18											
19											
20											
		Total	5	Ø	Ø	6	Ø	Ø	1	Ø	Ø

Reaction Description		
B-bruise	R-red	E-edema
B-significant bruise	R-significant redness	E-significant edema
< B-small/diminishing bruise	<R-slight/diminishing redness	<E-diminishing edema
B>-large/increasing bruise	R>-intense/increasing redness	E>-increasing edema

TST Reaction Form

Room: C5 Source: Mu Species: Cynos Group#: 05182017

Flashlight: Yes / No Total # animals in group: 97

			Date/Time/Initial 23May17 1045A (WP)			Date/Time/Initial 24May17 745A (WP)			Date/Time/Initial 25May17 Noon (WP)		
			24 hr Reaction			48 hr Reaction			72 hr Reaction		
	Cage#	Animal#	Bruise	Red	Edema	Bruise	Red	Edema	Bruise	Red	Edema
1	29 (M)	[redacted]	B	J		B	—		B	R	E.
2	30 (M)	[redacted]	<B	J		<B	—		<B	—	
3	31 (M)	[redacted]	<B	J		<B	—		—	—	
4	186 (F)	[redacted]	—	J		B	—		<B	—	
5	39 (M)	[redacted]	—	J		<B	—		—	—	
6	25 (M)	[redacted]		J				EB	<B	—	
7											
8											
9											
10											
11											
12											
13											
14											
15											
16											
17											
18											
19											
20											
		Total	3	—		5	—				

Reaction Description		
B-bruise	R-red	E-edema
B-significant bruise	R-significant redness	E-significant edema
< B-small/diminishing bruise	<R-slight/diminishing redness	<E-diminishing edema
B>-large/increasing bruise	R>-intense/increasing redness	E>-increasing edema

TST Reaction Form

Room: C3   Source: Mu   Species: Cynos   Group#: 05182017

Flashlight: Yes / No (Yes circled)   Total # animals in group: 97

			Date/Time/Initial 23 May 17 1035A [illegible]			Date/Time/Initial 24 May 17 8A [illegible]			Date/Time/Initial 25 May 17 1141A [illegible]		
			24 hr Reaction			48 hr Reaction			72 hr Reaction		
	Cage#	Animal#	Bruise	Red	Edema	Bruise	Red	Edema	Bruise	Red	Edema
1	9 (F)	[redacted]	<B	Ad01		<B	—	—	<B	—	—
2	12 (F)	[redacted]	CB	Ad01		<B	—	—	—	—	
3	19 (F)	[redacted]	<B	Ad01		<B	—	—	—	—	—
4	20 (F)	[redacted]	<B	Ad01		CB	—	—	—	—	
5	29 (F)	[redacted]	B	Ad01		—	—	—	—	—	—
6	30 (F)	[redacted]	B	Ad01		<B.	—	—	CB	—	—
7	41 (M)	[redacted]	B.	J		<B	—	—	—	—	—
8	48 (M)	[redacted]	B	J	—	<B	+ —	—	CB	—	—
9	34 (M)	[redacted]		J		<B	—	—	<B	—	—
10	39 (M)	[redacted]		J		CB	—	—	<B	—	—
11	40 (M)	[redacted]		J		CB	—	—	<B	—	—
12	44 (M)	[redacted]		J		CB	—	—	CB	—	—
13	46 (M)	[redacted]		J.		<B	—	—	CB.	—	—
14											
15											
16											
17											
18											
19											
20											
		Total	8	0	0	12	0	0	8	0	0

Reaction Description		
B-bruise	R-red	E-edema
B-significant bruise	R-significant redness	E-significant edema
< B-small/diminishing bruise	<R-slight/diminishing redness	<E-diminishing edema
B>-large/increasing bruise	R>-intense/increasing redness	E>-increasing edema
