# Supplementary material for: A retrospective analysis of the tuberculin skin test reactions of a single source population of Mauritian Macaca fascicularis held in quarantine during 2017
Source: PLoS One. 2022 Apr 14;17(4):e0265942. doi: 10.1371/journal.pone.0265942 (PMC9009605; doi:10.1371/journal.pone.0265942)
Supplement: S6 Dataset — (PDF) [file pone.0265942.s006.pdf]

TST Reaction Form

Room: C4 Source: MU Species: CY Group#: 07272017

Flashlight: Yes / (No) Total # animals in group: 89

			Date/Time/Initial 8/29/2017 7:48 [illegible]			Date/Time/Initial 8/30/17 16:25 [illegible]			Date/Time/Initial 8/31/17 13:47 [illegible]		
			24 hr Reaction			48 hr Reaction			72 hr Reaction		
	Cage#	Animal#	Bruise	Red	Edema	Bruise	Red	Edema	Bruise	Red	Edema
1	39(M)	[redacted]	<B	(J)		—			—		
2											
3											
4											
5											
6											
7											
8											
9											
10											
11											
12											
13											
14											
15											
16											
17											
18											
19											
20											
		Total	1	Ø	Ø	Ø	Ø	Ø	Ø	Ø	Ø

Br / 10M.

Reaction Description		
B-bruise	R-red	E-edema
B-significant bruise	R-significant redness	E-significant edema
< B-small/diminishing bruise	<R-slight/diminishing redness	<E-diminishing edema
B>-large/increasing bruise	R>-intense/increasing redness	E>-increasing edema

TST Reaction Form

Room: CS Source: MU Species: Cy Group#: 07272017

Flashlight: (Yes) / No Total # animals in group: 89

			Date/Time/Initial 8/29/17 17:40 [initials]			Date/Time/Initial 8/30/17 16:10 [initials]			Date/Time/Initial 8/31/17 13:35 [initials]		
			24 hr Reaction			48 hr Reaction			72 hr Reaction		
	Cage#	Animal#	Bruise	Red	Edema	Bruise	Red	Edema	Bruise	Red	Edema
1	5 (M)	[redacted]	B	(J)		B			<B		
2	6 (M)	[redacted]	<B	(J)		—			—		
3	15 (M)	[redacted]	<B	(J)		<<B			—		
4	16 (M)	[redacted]	<B	(J)		<B			<<B		
5	37 (F)	[redacted]	B	(J)		<B			<<B		
6											
7											
8											
9											
10											
11											
12											
13											
14											
15											
16											
17											
18											
19											
20											
		Total	5	Ø	Ø	4	Ø	Ø	3	Ø	Ø

89 / 40M / 13F

Reaction Description		
B-bruise	R-red	E-edema
B-significant bruise	R-significant redness	E-significant edema
< B-small/diminishing bruise	<R-slight/diminishing redness	<E-diminishing edema
B>-large/increasing bruise	R>-intense/increasing redness	E>-increasing edema

TST Reaction Form

Room: C4 Source: MW Species: Cyno Group#: 07272017

Flashlight: (Yes) / No 89 Total # animals in group: ~~37~~ ~~room~~ 89

			Date/Time/Initial 8/15/17 18:55 [illegible]			Date/Time/Initial 8/16/17 17:20 [illegible]			Date/Time/Initial 8/17/17 18:40 [illegible]		
			24 hr Reaction			48 hr Reaction			72 hr Reaction		
	Cage#	Animal#	Bruise	Red	Edema	Bruise	Red	Edema	Bruise	Red	Edema
1			—			—			—		
2											
3											
4											
5											
6											
7											
8											
9											
10											
11											
12											
13											
14											
15											
16											
17											
18											
19											
20											
		Total	Ø	Ø	Ø	Ø	Ø	Ø	Ø	Ø	Ø

Reaction Description		
B-bruise	R-red	E-edema
B-significant bruise	R-significant redness	E-significant edema
< B-small/diminishing bruise	<R-slight/diminishing redness	<E-diminishing edema
B>-large/increasing bruise	R>-intense/increasing redness	E>-increasing edema

TST Reaction Form

Room: C5 Source: MW Species: Cyn Group#: 07272017

Flashlight: Yes / No (Yes circled) 89 Total # animals in group: ~~52~~ 89

			Date/Time/Initial 8/15/17 18:40 [illegible]			Date/Time/Initial 8/16/17 17:10 [illegible]			Date/Time/Initial 8/17/17 18:20 [illegible]		
			24 hr Reaction			48 hr Reaction			72 hr Reaction		
	Cage#	Animal#	Bruise	Red	Edema	Bruise	Red	Edema	Bruise	Red	Edema
1	8 (M)	[redacted]	<B	(J)		<B			—		
2	26 (F)	[redacted]	B	(J)		<B			—		
3	37 (F)	[redacted]	B	(J)		<B			—		
4		[redacted]									
5											
6											
7											
8						BF					
9						1JM					
10						2JF					
11											
12											
13											
14											
15											
16											
17											
18											
19											
20											
		Total	3	∅	∅	3	∅	∅	∅	∅	∅

Reaction Description		
B-bruise	R-red	E-edema
<u>B</u>-significant bruise	<u>R</u>-significant redness	<u>E</u>-significant edema
< B-small/diminishing bruise	<R-slight/diminishing redness	<E-diminishing edema
B>-large/increasing bruise	R>-intense/increasing redness	E>-increasing edema

TST Reaction Form

Room: C5 Source: MU Species: Cepus Group#: 07272017

Flashlight: (Yes) / No Total # animals in group: 89

			Date/Time/Initial 1Aug17 ~10AM MP			Date/Time/Initial 2Aug17 ~11AM MP			Date/Time/Initial 3Aug17 ~10AM MP		
			24 hr Reaction			48 hr Reaction			72 hr Reaction		
	Cage#	Animal#	Bruise	Red	Edema	Bruise	Red	Edema	Bruise	Red	Edema
1	1 (M)	[redacted]	B	(J) —		<B	—		<B		—
2	4 (M)	[redacted]	<B	(J) —		<B	—		<B		—
3	8 (M)	[redacted]	B	(J) —		B	—		<B		—
4	16 (M)	[redacted]	B	(J) —		<B	—		<B		
5	21 (M)	[redacted]	B	(J) —		<B	—		—		
6	24 (F)	[redacted]	<B	(J) —		<B	—		—		
7	26 (F)	[redacted]	—	(J) —		<B	—		<B	—	
8	30 (F)	[redacted]	—	(J) —		B	—		<B	—	
9	36 (F)	[redacted]	B	(J) —		<B	—		<B	—	
10	37 (F)	[redacted]	B	(J) —		B	—		<B		—
11											
12											
13	1	[redacted] (J) (J)				SJM					
14											
15						SJF.					
16											
17											
18											
19											
20											
		Total	8	—		10	—		8		—

Reaction Description		
B-bruise	R-red	E-edema
B-significant bruise	R-significant redness	E-significant edema
< B-small/diminishing bruise	<R-slight/diminishing redness	<E-diminishing edema
B>-large/increasing bruise	R>-intense/increasing redness	E>-increasing edema

TST Reaction Form

Room: C4 Source: MU Species: cepus Group#: 07272017

Flashlight: (Yes) No Total # animals in group: 89

			Date/Time/Initial 1Aug17 240AMP			Date/Time/Initial 2Aug17 211AMP			Date/Time/Initial 3Aug17 240AMP		
			24 hr Reaction			48 hr Reaction			72 hr Reaction		
	Cage#	Animal#	Bruise	Red	Edema	Bruise	Red	Edema	Bruise	Red	Edema
1	2 (M)	[redacted]		(Adult)	—	<B	—		<B	—	
2	20 (M)	[redacted]	B	(Adult)	—	<B	—		—		
3	21 (M)	[redacted]	B	(Adult)		<B	—		—		
4	25 (M)	[redacted]	B	(Adult)		<B	—		<B	—	
5	27 (M)	[redacted]	B	(Adult)		<B	—		—		
6	34 (M)	[redacted]	B	(Adult)		—	—		—		
7	37 (M)	[redacted]	<B	(J)	—		—		—		
8	39 (M)	[redacted]	<B	(J)	—		—		—		
9											
10											
11											
12											
13											
14											
15											
16											
17											
18											
19											
20											
		Total	7	—		5	—		2	—	

2JM
1 Adult M.
5 Adult M

Reaction Description		
B-bruise	R-red	E-edema
B-significant bruise	R-significant redness	E-significant edema
< B-small/diminishing bruise	<R-slight/diminishing redness	<E-diminishing edema
B>-large/increasing bruise	R>-intense/increasing redness	E>-increasing edema
