# Supplementary material for: A retrospective analysis of the tuberculin skin test reactions of a single source population of Mauritian Macaca fascicularis held in quarantine during 2017
Source: PLoS One. 2022 Apr 14;17(4):e0265942. doi: 10.1371/journal.pone.0265942 (PMC9009605; doi:10.1371/journal.pone.0265942)
Supplement: S7 Dataset — (PDF) [file pone.0265942.s007.pdf]

TST Reaction Form

Room: C2 Source: MU Species: Cynos Group#: 0831 2017

Flashlight: (Yes) / No Total # animals in group: 107

	Cage#	Animal#	24 hr Reaction<br>Date/Time/Initial<br>11/7/17 17:20			48 hr Reaction<br>Date/Time/Initial<br>17:10 11/8/17			72 hr Reaction<br>Date/Time/Initial<br>11/9/17 17:40		
			Bruise	Red	Edema	Bruise	Red	Edema	Bruise	Red	Edema
1	1 (F)	(X)	<B			<B			–		
2	1 (F)	(X)	<B			–			–		
3	19 (M)	(J)	<B			<<B			–		
4	40 (M)	(J)	<B			<<B			<<B		
5	11 (M)	(J)				<B			–		
6											
7											
8											
9											
10											
11											
12											
13											
14											
15											
16											
17											
18											
19											
20											
		Total	4	Ø	Ø	4	Ø	Ø	1	Ø	Ø

BR
2F
3M.

Reaction Description		
B-bruise	R-red	E-edema
<u>B</u>-significant bruise	<u>R</u>-significant redness	<u>E</u>-significant edema
< B-small/diminishing bruise	<R-slight/diminishing redness	<E-diminishing edema
B>-large/increasing bruise	R>-intense/increasing redness	E>-increasing edema

TST Reaction Form

Room: C2 Source: MU Species: Cynos Group#: 08312017

Flashlight: (Yes) / No Total # animals in group: ~~105~~ 107

			Date/Time/Initial 5Sep17 440P			Date/Time/Initial 6Sep17 255P			Date/Time/Initial 7Sep17 830AM		
			24 hr Reaction			48 hr Reaction			72 hr Reaction		
	Cage#	Animal#	Bruise	Red	Edema	Bruise	Red	Edema	Bruise	Red	Edema
1	2 (F)		✓	(J) —		<	—		<	—	
2	6 (M)		✓	(J) —		<	—		<	—	
3	7 (M)		<	(J) —		<	—		<	—	
4	8 (M)		<	(J)	[illegible]	<	—		<	—	
5	10 (M)		—	(J)	(E)	—		mod E	[illegible]	< [illegible] E	
6	11 (M)		<	(J) —		<	—		—	—	
7	13 (M)		<	(J) —		<	—		<	—	
8	20 (M)		<	(J) —		<	—		—	—	
9	36 (M)	(J)	<	—		<	—		<	—	
10	39 (M)	(J)	<	—		<	—		—	—	
11	40 (M)	(J)	—	—	—	—	—		<	—	
12											
13											
14											
15											
16											
17											
18											
19											
20											
		Total	9	0	1						

1JT Br. 9/8JM.

Ed / 1JM.

Reaction Description		
B-bruise	R-red	E-edema
B-significant bruise	R-significant redness	E-significant edema
< B-small/diminishing bruise	<R-slight/diminishing redness	<E-diminishing edema
B>-large/increasing bruise	R>-intense/increasing redness	E>-increasing edema

TST Reaction Form

Room: C1 Source: MU Species: Cynos Group#: 08312017

Flashlight: Yes / No Total # animals in group: ~~105~~ 107

			Date/Time/Initial<br>5Sep17 2:00PM MP			Date/Time/Initial<br>6Sep17 2:00PM MP			Date/Time/Initial<br>7Sep17		
			24 hr Reaction			48 hr Reaction			72 hr Reaction		
	Cage#	Animal#	Bruise	Red	Edema	Bruise	Red	Edema	Bruise	Red	Edema
1	18 (F)	(J)		—	—	<B	—	—	<B	—	—
2	16 (F)	(J)		—	—	—	—		<B	—	—
3											
4		8F									
5		2J F									
6											
7											
8											
9											
10											
11											
12											
13											
14											
15											
16											
17											
18											
19											
20											
		Total	—	—	—	1	—		2	—	

Reaction Description		
B-bruise	R-red	E-edema
<u>B</u>-significant bruise	<u>R</u>-significant redness	<u>E</u>-significant edema
< B-small/diminishing bruise	<R-slight/diminishing redness	<E-diminishing edema
B>-large/increasing bruise	R>-intense/increasing redness	E>-increasing edema

13 Sept 1410 PM 14 Sep 810 AM

yes light

12 Sep 5PM

Br

8 JF

13 JM

3 Adol M.

C2

6 (M) (J) B|CB|KB
(M) J

7 (M) B|C B|CB
9 (M) 24-|CB|CB
B|C B|KB
12 (M) (J)
15 (M) B|CB|KB
(J)
16 (M) B|C B|CB
(J) <B|CB|CB
(M) B|CB
19 (M) (J) C B|CB|—
20 (M) B|KB
2(M) CB|CB|CB
27 (M)

29 (M) CB|CB|—

34 (M) BKB|— (J)
35 (M) B|CB|CB (J) (M) J B|CB
36 (M) (F) B|CB|KB

13

C1

(F) CB|KB|— (J)
(F) CB|KB|— (J)
3 (F) C B|KB|KB (J)
8
9 (F) CB|KB|— (J)
10 (F) 3|CB|CB (J)
12 (F) CB|KB|KB (J)
16 (F) CB|CB|CB (J)
17 (F) CB|CB|CB (J)

(Adol)
(Adol)
(Adol)

TST Reaction Form

Room: C-1-2 Source: MU Species: Cyno Group#: 083/2017

Flashlight: (Yes) / No RE Total # animals in group: 107

			Date/Time/Initial 1500 24Oct17 MP			Date/Time/Initial 1540 25Oct17 MP			Date/Time/Initial 1505 26Oct17 MP		
			24 hr Reaction			48 hr Reaction			72 hr Reaction		
	Cage#	Animal#	Bruise	Red	Edema	Bruise	Red	Edema	Bruise	Red	Edema
1	35 (M)	(J)	—	—	—	CB	—		CB	—	
2	33 (M)	J	—	—	—	CB	—		CB	—	
3	20 (M)	(J)				CB	—		—	—	—
4	12 (M)	(J)		—	—	B	—	—	CB	—	
5	9 (M)	(J)		—	—	CB	—		CB	—	—
6	(M)	(J)	—	—	—	—	—		CB	—	
7											
8											
9											
10											
11											
12											
13											
14											
15											
16											
17											
18											
19											
20											
		Total	Ø	Ø	Ø	5	Ø	Ø	5	Ø	Ø

BF / 6JM

Reaction Description		
B-bruise	R-red	E-edema
B-significant bruise	R-significant redness	E-significant edema
< B-small/diminishing bruise	<R-slight/diminishing redness	<E-diminishing edema
B>-large/increasing bruise	R>-intense/increasing redness	E>-increasing edema

AC12

TST Reaction Form

Room: C6 Source: mu Species: Cynos Group#: 08312017

Flashlight: (Yes) / No Total # animals in group: 107

			Date/Time/Initial 11/7/17 17:39			Date/Time/Initial 11/8/17 17:25			Date/Time/Initial 11/9/17 17:45		
C12			24 hr Reaction			48 hr Reaction			72 hr Reaction		
#	Cage#	Animal#	Bruise	Red	Edema	Bruise	Red	Edema	Bruise	Red	Edema
1	153 (F)	[illegible] (J)	<B			<<B			–		
2	207 (F)	[illegible] (J)	<B			<<B			–		
3	2714 (F)	[illegible] (J)	<B			–			–		
4	21 9 (F)	[illegible] (J)				<B			–		
5											
6											
7											
8											
9											
10											
11											
12											
13											
14											
15											
16											
17											
18											
19											
20											
		Total	3	Ø	Ø	3	Ø	Ø	Ø	Ø	Ø

Reaction Description		
B-bruise	R-red	E-edema
B-significant bruise	R-significant redness	E-significant edema
< B-small/diminishing bruise	<R-slight/diminishing redness	<E-diminishing edema
B>-large/increasing bruise	R>-intense/increasing redness	E>-increasing edema

TST Reaction Form

Room: C?1 Source: MY Species: Cyno Group#: 083/2017

Flashlight: (Yes) / No RG Total # animals in group: 107

			Date/Time/Initial<br>1445 24Oct17 MP			Date/Time/Initial<br>1545 25Oct17 MP			Date/Time/Initial<br>1435 26Oct17 MP		
			24 hr Reaction			48 hr Reaction			72 hr Reaction		
	Cage#	Animal#	Bruise	Red	Edema	Bruise	Red	Edema	Bruise	Red	Edema
1	18	Both	—	—	—	<B	—	—	<B	—	—
2		(F)	(F)								
3		(F)	(F)								
4											
5											
6											
7											
8				BR							
9											
10				2JF							
11											
12											
13											
14											
15											
16											
17											
18											
19											
20											
		Total	Ø	Ø	Ø	2	Ø	Ø	2	Ø	Ø

Reaction Description		
B-bruise	R-red	E-edema
B-significant bruise	R-significant redness	E-significant edema
< B-small/diminishing bruise	<R-slight/diminishing redness	<E-diminishing edema
B>-large/increasing bruise	R>-intense/increasing redness	E>-increasing edema

TST Reaction Form

Room: C1 Source: MN Species: cyno Group#: 08312015

Flashlight: (Yes) / No Total # animals in group: 107

			Date/Time/Initial 10Oct17 1340 MP			Date/Time/Initial 11Oct17 1500 MP			Date/Time/Initial 1400 12Oct17 MP		
			24 hr Reaction			48 hr Reaction			72 hr Reaction		
	Cage#	Animal#	Bruise	Red	Edema	Bruise	Red	Edema	Bruise	Red	Edema
1	3 (F)	[illegible] (J)	<B	—		—	—		—	—	
2	7 (F)	[illegible] (J)	<B	—		<B	—		<B	—	
3	13 (F)	[illegible] (J)	<B	—		—	—		—	—	
4	16 (F)	[illegible] (J)	<B	—		—	—		—	—	
5											
6											
7				KF							
8				LWF							
9											
10											
11											
12											
13											
14											
15											
16											
17											
18											
19											
20											
		Total	4	—		1	—		1	—	

Reaction Description		
B-bruise	R-red	E-edema
B-significant bruise	R-significant redness	E-significant edema
< B-small/diminishing bruise	<R-slight/diminishing redness	<E-diminishing edema
B>-large/increasing bruise	R>-intense/increasing redness	E>-increasing edema

TST Reaction Form

Room: C1 Source: MU. Species: Cy Group#: 08312017

Flashlight: (Yes) / No Total # animals in group: 105

			Date/Time/Initial 9/26/17 17:55 [initials]			Date/Time/Initial 9/27/17 19:40 [initials]			Date/Time/Initial 9/28/17 20:20 [initials]		
			24 hr Reaction			48 hr Reaction			72 hr Reaction		
	Cage#	Animal#	Bruise	Red	Edema	Bruise	Red	Edema	Bruise	Red	Edema
1	3 (F)	[illegible] (5)	B			<<B			—		
2	10 (F)	[illegible] (6)				<B			—		
3											
4											
5											
6					B8/2JF						
7											
8											
9											
10											
11											
12											
13											
14											
15											
16											
17											
18											
19											
20											
		Total	1	Ø	Ø	2	Ø	Ø	Ø	Ø	Ø

Reaction Description		
B-bruise	R-red	E-edema
B-significant bruise	R-significant redness	E-significant edema
< B-small/diminishing bruise	<R-slight/diminishing redness	<E-diminishing edema
B>-large/increasing bruise	R>-intense/increasing redness	E>-increasing edema

TST Reaction Form

Room: C2 Source: MW LE Species: Cyno. Group#: 083/2017

Flashlight: (Yes) / No Total # animals in group: 107

			Date/Time/Initial 10Oct17 1347 MP.			Date/Time/Initial 11Oct17 1448 MP			Date/Time/Initial 12Oct17 1405 MP		
			24 hr Reaction			48 hr Reaction			72 hr Reaction		
	Cage#	Animal#	Bruise	Red	Edema	Bruise	Red	Edema	Bruise	Red	Edema
1	2	[illegible] (F)	(J) B	—		<B	—		—	—	
2		(F)	(J) <B	—		—	—		—	—	
3	3	(F)	(J) B	—		<B	—		<B	—	
4	5	(F)	(J) <B	—		<B	—		<B	—	
5	6	(M)	(J) <B	—		<B	—		<B	—	
6	7	(M)	(J) <B	—		<B	—		<B	—	
7	9	(M)	(J) <B	—		<B	—		<B	—	
8	12	(M)	(J) <B	—		<B	—		—	—	
9	16	(M)	(J) <B	—		<B	—		<B	—	
10	27	(M)	(Adol) <B	—		<B	—		—	—	
11	34	(M)	(J) <B	—		<B	—		<B	—	
12	38	(M)	(J) <B	—		<B	—		<B	—	
13	41	(M)	(J) <B	—		<B	—		—	—	
14	40	(M)	(J) —	—	—	<B	—		<B	—	
15	35	(M)	(J) —	—	—	<B	—		<B	—	—
16	18	(M)	(J) —	—		<B	—	—	<B	—	
17		(M)	(J) —	—		<B	—		<B	—	
18	12	(M)	(J) —	—		<B	—		—	—	
19											
20											
		Total	13	Ø	Ø	17	Ø	Ø	12	—	—

Reaction Description		
B-bruise	R-red	E-edema
B-significant bruise	R-significant redness	E-significant edema
< B-small/diminishing bruise	<R-slight/diminishing redness	<E-diminishing edema
B>-large/increasing bruise	R>-intense/increasing redness	E>-increasing edema

Br. 4JF 13JM Adol 1M.

TST Reaction Form

Room: C2 Source: MU Species: Cy Group#: 08312017

Flashlight: (Yes) / No Total # animals in group: ~~105~~ 107

			Date/Time/Initial 9/26/17 17:40			Date/Time/Initial 9/27/17 19:25			Date/Time/Initial 9/28/17 20:10		
			24 hr Reaction			48 hr Reaction			72 hr Reaction		
	Cage#	Animal#	Bruise	Red	Edema	Bruise	Red	Edema	Bruise	Red	Edema
1	8 (M)	[illegible] (J)	<B			<			—		
2	18 (M)	[illegible] (J)	<B			—			—		
3	32 (M)	[illegible] (J)	B			<B			—		
4	35 (M)	[illegible] (J)	B			<B			—		
5											
6											
7											
8											
9											
10											
11											
12											
13											
14											
15											
16											
17											
18											
19											
20											
		Total	4	Ø	Ø	2	Ø	Ø	Ø	Ø	Ø

Br / 4JM

Reaction Description		
B-bruise	R-red	E-edema
B-significant bruise	R-significant redness	E-significant edema
< B-small/diminishing bruise	<R-slight/diminishing redness	<E-diminishing edema
B>-large/increasing bruise	R>-intense/increasing redness	E>-increasing edema
