# Supplementary material for: A retrospective analysis of the tuberculin skin test reactions of a single source population of Mauritian Macaca fascicularis held in quarantine during 2017
Source: PLoS One. 2022 Apr 14;17(4):e0265942. doi: 10.1371/journal.pone.0265942 (PMC9009605; doi:10.1371/journal.pone.0265942)
Supplement: S8 Dataset — (PDF) [file pone.0265942.s008.pdf]

TST Reaction Form

Room: CS Source: Mu Species: Cyno Group#: 09282017

Flashlight: Yes / No LE Total # animals in group: 106

			Date/Time/Initial 12/5/17 17:20 [initial]			Date/Time/Initial 12/6/17 16:40 [initial]			Date/Time/Initial 12/7/17 17:23 [initial]		
			24 hr Reaction			48 hr. Reaction			72 hr Reaction		
	Cage#	Animal#	Bruise	Red	Edema	Bruise	Red	Edema	Bruise	Red	Edema
1	2 (F)	[illegible] (J)	<<B			—					
2	3 (F)	[illegible] (J)	<B			<<B			—		
3	38 (F)	[illegible] (J)	<B			<B			<<B		
4											
5				Br							
6											
7				3JF.							
8											
9											
10											
11											
12											
13											
14											
15											
16											
17											
18											
19											
20											
		Total		3	Ø	Ø	2	Ø	Ø	Ø	Ø

Reaction Description		
B-bruise	R-red	E-edema
B-significant bruise	R-significant redness	E-significant edema
< B-small/diminishing bruise	<R-slight/diminishing redness	<E-diminishing edema
B>-large/increasing bruise	R>-intense/increasing redness	E>-increasing edema

TST Reaction Form

Room: C4 Source: Mu Species: Cyno Group#: 09282017

Flashlight: Yes / No LE Total # animals in group: 106

			Date/Time/Initial 12/5/17 17:40 [illegible]			Date/Time/Initial 12/6/17 16:50 [illegible]			Date/Time/Initial 12/7/17 17:40 [illegible]		
			24 hr Reaction			48 hr Reaction			72 hr Reaction		
	Cage#	Animal#	Bruise	Red	Edema	Bruise	Red	Edema	Bruise	Red	Edema
1	5 (M)	(J)	<B			<<B			—		
2	15 (M)	(J)	<B			<<B			—		
3	27 (M)	(Adol)	<B			<<B			—		
4											
5											
6											
7											
8											
9											
10											
11											
12											
13											
14											
15											
16											
17											
18											
19											
20											
		Total	3	Ø	Ø	3	Ø	Ø	Ø	Ø	Ø

BC
2JM
1AdolM

Reaction Description		
B-bruise	R-red	E-edema
B-significant bruise	R-significant redness	E-significant edema
< B-small/diminishing bruise	<R-slight/diminishing redness	<E-diminishing edema
B>-large/increasing bruise	R>-intense/increasing redness	E>-increasing edema

TST Reaction Form

Room: C4 Source: MU Species: Cynos Group#: 09282017

Flashlight: Yes / No RE Total # animals in group: 106

	Cage#	Animal#	Date/Time/Initial 11/23/17 20:15			Date/Time/Initial 11/23/17 17:20			Date/Time/Initial 11/24/17 17:12		
			24 hr Reaction			48 hr Reaction			72 hr Reaction		
			Bruise	Red	Edema	Bruise	Red	Edema	Bruise	Red	Edema
1	13 (M)	(J)	<B			<<B			–		
2											
3			KB1 / 1JM								
4											
5											
6											
7											
8											
9											
10											
11											
12											
13											
14											
15											
16											
17											
18											
19											
20											
		Total	1	Ø	Ø	1	Ø	Ø	Ø	Ø	Ø

Reaction Description		
B-bruise	R-red	E-edema
B-significant bruise	R-significant redness	E-significant edema
< B-small/diminishing bruise	<R-slight/diminishing redness	<E-diminishing edema
B>-large/increasing bruise	R>-intense/increasing redness	E>-increasing edema

TST Reaction Form

Room: CS Source: Mu Species: Cynos Group#: 09282017

Flashlight: Yes / No (circled: Yes) RE Total # animals in group: 106

			Date/Time/Initial 11/23/17 20:00 [initials]			Date/Time/Initial 11/24/17 17:10 [initials]			Date/Time/Initial 11/24/17 17:00 [initials]		
			24 hr Reaction			48 hr Reaction			72 hr Reaction		
	Cage#	Animal#	Bruise	Red	Edema	Bruise	Red	Edema	Bruise	Red	Edema
1	1 (F)	(J)	<B			<<B			–		
2	39 (F)	(J)	<B			<<B			–		
3	6 (F)	(J)	[illegible]			<<B			–		
4											
5											
6			Br								
7			—								
8			BJF								
9											
10											
11											
12											
13											
14											
15											
16											
17											
18											
19											
20											
		Total	2	Ø	Ø	3	Ø	Ø	Ø	Ø	Ø

Reaction Description		
B-bruise	R-red	E-edema
B-significant bruise	R-significant redness	E-significant edema
< B-small/diminishing bruise	<R-slight/diminishing redness	<E-diminishing edema
B>-large/increasing bruise	R>-intense/increasing redness	E>-increasing edema

TST Reaction Form

Room: C4 Source: MU Species: Cyno Group#: 09282017

Flashlight: (Yes) / No LE Total # animals in group: 106

			Date/Time/Initial 1400 8Nov17 MP			Date/Time/Initial 1427 9Nov17 MP			Date/Time/Initial 1545 10Nov17 MP		
			24 hr Reaction			48 hr Reaction			72 hr Reaction		
	Cage#	Animal#	Bruise	Red	Edema	Bruise	Red	Edema	Bruise	Red	Edema
1	3 (M)	(J)	<B	—		<B	—		—	—	
2	7 (M)	(J)	<B	—		<B	—		<B	—	
3	8 (M)	(J)	<B	—		<B	—		<B	—	
4	(M)	(J)	<B	—		<B	—		<B	—	
5	11 (M)	(J)	B	—		<B	—		<B	—	
6	12 (M)	(J)	B	—		<B	—		<B	—	
7	(M)	C (J)	<B	—		<B	—		<B	—	
8	24 (M)	(Adult)	<B	—		<B	—		—	—	
9	30 (M)	(Adult)	B	—		B	—		<B	—	
10	32 (M)	(J)	<B	—		<B	—		<B	—	
11	(M)	(J)	<B	—		<B	—		<B	—	
12	33 (M)	(J)	B	—		<B	—		<B	—	
13	37 (M)	(J)	<B	—		<B	—		<B	—	
14	38 (M)	(J)	<B	—		<B	—		<B	—	
15	15 (M)	(J)	—	—		<B	—		<B	—	
16	1 (M)	(J)		—		<B	—		<B	—	
17											
18											
19											
20											
		Total	14	Ø	Ø	16	Ø	Ø	14	Ø	Ø

Br
14JM
1Adult M
1Adult M

Reaction Description		
B-bruise	R-red	E-edema
B-significant bruise	R-significant redness	E-significant edema
< B-small/diminishing bruise	<R-slight/diminishing redness	<E-diminishing edema
B>-large/increasing bruise	R>-intense/increasing redness	E>-increasing edema

TST Reaction Form

Room: CS Source: MU Species: Cyno Group#: 09282017

Flashlight: (Yes) / No LE Total # animals in group: 106

	Cage#	Animal#	Date/Time/Initial 1412 8NOV17 MP<br>24 hr Reaction Bruise	Red	Edema	Date/Time/Initial 1412 9NOV17 MP<br>48 hr Reaction Bruise	Red	Edema	Date/Time/Initial 1355 10NOV2017 MP<br>72 hr Reaction Bruise	Red	Edema
1	5 (F)	(J)	<B	—		<B	—			—	
2	6 (F)	(J)	<B	—		<B	—			—	
3	(F)	(J)	<B	—		<B	—		<B	—	
4	31 (F)	(J)	<B	—		<B	—		—		
5	32 (F)	(J)	<B	—		<B	—		<B	—	
6	33 (F)	(J)	B	—		<B	—		<B	—	
7	40 (F)	(J)	<B	—		<B	—		<B	—	
8	37 (F)	(J)		—		B	—		<B	—	
9											
10			Br								
11											
12											
13			8JF								
14											
15											
16											
17											
18											
19											
20											
		Total	7	Ø	Ø	6	Ø	Ø	5	Ø	Ø

Reaction Description		
B-bruise	R-red	E-edema
B-significant bruise	R-significant redness	E-significant edema
< B-small/diminishing bruise	<R-slight/diminishing redness	<E-diminishing edema
B>-large/increasing bruise	R>-intense/increasing redness	E>-increasing edema

TST Reaction Form

Room: C4 Source: mu Species: Cyno Group#: 09282017

Flashlight: Yes / No (Yes circled) Total # animals in group: 106

			Date/Time/Initial 10/25/17 14:32 [illegible]			Date/Time/Initial 10/26/17 1515 [illegible]			Date/Time/Initial 10/27/17 1942 [illegible]		
			24 hr Reaction			48 hr Reaction			72 hr Reaction		
	Cage#	Animal#	Bruise	Red	Edema	Bruise	Red	Edema	Bruise	Red	Edema
1	5	Ⓜ [illegible] Ⓙ	B			B			<B		
2	8	Ⓜ [illegible] Ⓙ	<B			—			—		
3		Ⓜ [illegible] Ⓙ	<B			<B			—		
4	12	Ⓜ [illegible] Ⓙ	<B			—			~		
5	18	Ⓜ [illegible] Ⓙ	<B			<B			<B		
6											
7											
8			KC								
9											
10			SJM.								
11											
12											
13											
14											
15											
16											
17											
18											
19											
20											
		Total	5	Ø	Ø	3	Ø	Ø	2	Ø	Ø

Reaction Description		
B-bruise	R-red	E-edema
B-significant bruise	R-significant redness	E-significant edema
< B-small/diminishing bruise	<R-slight/diminishing redness	<E-diminishing edema
B>-large/increasing bruise	R>-intense/increasing redness	E>-increasing edema

TST Reaction Form

Room: CS Source: mu Species: Cyno Group#: 09282017

Flashlight: Yes / No (Yes circled) Total # animals in group: 106

		RT	Date/Time/Initial 10/25/17 14:10 [initials] 24 hr Reaction			Date/Time/Initial 10/26/17 14.55 [initials] 48 hr Reaction			Date/Time/Initial 10/27/17 19:30 [initials] 72 hr Reaction		
	Cage#	Animal#	Bruise	Red	Edema	Bruise	Red	Edema	Bruise	Red	Edema
1	5 (F)	(J)	<B			<B			–		
2		(J)	<B			–			–		
3	6 (F)	(J)	<B			<<B			–		
4	34 (F)	(J)	<B			<B			–		
5	37 (F)	(J)	B			<B			<<B		
6											
7											
8			Br								
9											
10			SJF								
11											
12											
13											
14											
15											
16											
17											
18											
19											
20											
		Total	5	Ø	Ø	4	Ø	Ø	1	Ø	Ø

Reaction Description		
B-bruise	R-red	E-edema
B-significant bruise	R-significant redness	E-significant edema
< B-small/diminishing bruise	<R-slight/diminishing redness	<E-diminishing edema
B>-large/increasing bruise	R>-intense/increasing redness	E>-increasing edema

cg84 bruise during BT.

TST Reaction Form

Room: C5 Source: MY Species: Cy Group#: 09282017

Flashlight: (Yes) / No Total # animals in group: 106

			Date/Time/Initial 10/11/17 1500 [illegible] 24 hr Reaction			Date/Time/Initial 10/12/17 15:10 [illegible] 48 hr Reaction			Date/Time/Initial 10/13/17 16:35 [illegible] 72 hr Reaction		
	Cage#	Animal#	Bruise	Red	Edema	Bruise	Red	Edema	Bruise	Red	Edema
1	8 (F)	[illegible] (J)	<B			<<B			—		
2	27 (F)	[illegible] (J)	B			<B			<		
3	31 (F)	[illegible] (J)	<B			—			—		
4	34 (F)	[illegible] (J)	<B			—			—		
5											
6											
7			Br								
8											
9											
10			4JF								
11											
12											
13											
14											
15											
16											
17											
18											
19											
20											
		Total	4	Ø	Ø	2	Ø	Ø	Ø	Ø	Ø

Reaction Description		
B-bruise	R-red	E-edema
B-significant bruise	R-significant redness	E-significant edema
< B-small/diminishing bruise	<R-slight/diminishing redness	<E-diminishing edema
B>-large/increasing bruise	R>-intense/increasing redness	E>-increasing edema

TST Reaction Form

Room: C5 Source: MU Species: cy Group#: 09282017

Flashlight: (Yes) / No Total # animals in group: 106

			Date/Time/Initial 3Oct17 1355 MP			Date/Time/Initial 4Oct17 1350 MP			Date/Time/Initial 5Oct17 0924 MP		
			24 hr Reaction			48 hr Reaction			72 hr Reaction		
	Cage#	Animal#	Bruise	Red	Edema	Bruise	Red	Edema	Bruise	Red	Edema
1	6 (F)	(J)	<B	—		<B	—		<B	—	
2	7 (F)	(J)	<B	—		<B	—			—	
3	10 (F)	(J)	B	—	E	—	R	E	—	R	E
4	34 (F)	(J)	B	—		<B	—		<B	—	
5	36 (F)	(J)	<B	—		<B	—		<B	—	
6	36 (F)	(J)				<B	—		<R	—	
7											
8											
9											
10											
11											
12											
13											
14											
15											
16											
17											
18											
19											
20											
		Total									

Br TJF

red / edema TJF

Reaction Description		
B-bruise	R-red	E-edema
B-significant bruise	R-significant redness	E-significant edema
< B-small/diminishing bruise	<R-slight/diminishing redness	<E-diminishing edema
B>-large/increasing bruise	R>-intense/increasing redness	E>-increasing edema

TST Reaction Form

Room: C4 Source: MU Species: Cy Group#: 09282017

Flashlight: (Yes) / No Total # animals in group: 106

			Date/Time/Initial 10/11/17 15:20 [initial]			Date/Time/Initial 10/12/17 15:25 [initial]			Date/Time/Initial 10/13/17 16:00 [initial]		
			24 hr Reaction			48 hr Reaction			72 hr Reaction		
	Cage#	Animal#	Bruise	Red	Edema	Bruise	Red	Edema	Bruise	Red	Edema
1	6 (M)	(J)	<B			<B			<<B		
2	16 (M)	(J)	<B			<<B			–		
3	18 (M)	(J)	B			B	–	+	<B	OK Rm	Vet CK
4	33 (M)	(J)	<B			<<B			–		
5	34 (M)	(J)	<B			–			–		
6	39 (M)	(J)	<B			–			–		
7	3 (M)	(J)				<B			<<B		
8	13 (M)	(J)				<B			<<B		
9											
10											
11			Br			Edema					
12											
13			JJM			LJM.					
14											
15											
16											
17											
18											
19											
20											
		Total	6	Ø	Ø	6	Ø	1	4	Ø	Ø

Reaction Description		
B-bruise	R-red	E-edema
B-significant bruise	R-significant redness	E-significant edema
< B-small/diminishing bruise	<R-slight/diminishing redness	<E-diminishing edema
B>-large/increasing bruise	R>-intense/increasing redness	E>-increasing edema

TST Reaction Form

Room: C4 Source: MV Species: Cy Group#: 09282017

Flashlight: Yes / No (Yes circled) Total # animals in group: 106

	Cage#	Animal#	24 hr Reaction Bruise	Red	Edema	48 hr Reaction Bruise	Red	Edema	72 hr Reaction Bruise	Red	Edema
			Date/Time/Initial 30ct17 1415 MP			Date/Time/Initial 40ct17 1335 MP			Date/Time/Initial 50ct17 0943 MP		
1	4 (M)	(J)	B	—		<B	—		—		
2	11 (M)	(J)	<B	—		<B	—		<B	—	
3	15 (M)	(J)	B	—		<B	—		<B	—	
4	16 (M)	(J)	B	—		<B	—		wt 5 43 <B	—	
5	32 (M)	(J)	<B	—		<B	—		—		
6	37 (M)	(J)	<B	—		<B	—		—		
7	38 (M)	(J)	<B	—		<B	—		<B	—	
8	27 (M)	(Adol)	—			<B	—		—		
9											
10											
11											
12											
13											
14											
15											
16											
17											
18											
19											
20											
		Total	7	—		9	—		4	—	

Br Adol

7JM 1M

Reaction Description		
B-bruise	R-red	E-edema
B-significant bruise	R-significant redness	E-significant edema
< B-small/diminishing bruise	<R-slight/diminishing redness	<E-diminishing edema
B>-large/increasing bruise	R>-intense/increasing redness	E>-increasing edema
