# Supplementary material for: A retrospective analysis of the tuberculin skin test reactions of a single source population of Mauritian Macaca fascicularis held in quarantine during 2017
Source: PLoS One. 2022 Apr 14;17(4):e0265942. doi: 10.1371/journal.pone.0265942 (PMC9009605; doi:10.1371/journal.pone.0265942)
Supplement: S9 Dataset — (PDF) [file pone.0265942.s009.pdf]

TST Reaction Form

Room: C1 Source: Mu Species: Cyno Group#: 12072017

Flashlight: (Yes) / No NE Total # animals in group: 98

			Date/Time/Initial 10Jan18 1610 MP			Date/Time/Initial 11Jan18 1210 MP			Date/Time/Initial 12Jan18 0830 MP		
			24 hr Reaction			48 hr Reaction			72 hr Reaction		
	Cage#	Animal#	Bruise	Red	Edema	Bruise	Red	Edema	Bruise	Red	Edema
1	12 (F)	(F)	B	—		<B	—	—	<B		—
2											
3											
4											
5											
6											
7											
8											
9											
10											
11											
12											
13											
14											
15											
16											
17											
18											
19											
20											
		Total	1	Ø	Ø	1	Ø	Ø	1	Ø	Ø

Br

1JF

Reaction Description		
B-bruise	R-red	E-edema
B-significant bruise	R-significant redness	E-significant edema
< B-small/diminishing bruise	<R-slight/diminishing redness	<E-diminishing edema
B>-large/increasing bruise	R>-intense/increasing redness	E>-increasing edema

TST Reaction Form

Room: C1 Source: MU Species: Cynos Group#: 12072017

Flashlight: Yes / No LC Total # animals in group: 98

	Cage#	Animal#	Date/Time/Initial 12/27/17 18:20 [illegible]			Date/Time/Initial 12/28/17 18:00 [illegible]			Date/Time/Initial 12/29/17 13:35 [illegible]		
			24 hr Reaction			48 hr Reaction			72 hr Reaction		
			Bruise	Red	Edema	Bruise	Red	Edema	Bruise	Red	Edema
1	19	[illegible]	<B			<B			<<B		
2	(F)		(J)								
3											
4											
5											
6				Br							
7											
8				IJF.							
9											
10											
11											
12											
13											
14											
15											
16											
17											
18											
19											
20											
		Total	1	Ø	Ø	1	Ø	Ø	1	Ø	Ø

Reaction Description		
B-bruise	R-red	E-edema
B-significant bruise	R-significant redness	E-significant edema
< B-small/diminishing bruise	<R-slight/diminishing redness	<E-diminishing edema
B>-large/increasing bruise	R>-intense/increasing redness	E>-increasing edema

TST Reaction Form

Room: C 2 Source: MU Species: Cynos Group#: 12072017

Flashlight: Yes / No Total # animals in group: 98

			Date/Time/Initial 12/27/17 18:10 [initials]			Date/Time/Initial 12/28/17 17:40 [initials]			Date/Time/Initial 12/29/17 15:20 [initials]		
			24 hr Reaction			48 hr Reaction			72 hr Reaction		
	Cage#	Animal#	Bruise	Red	Edema	Bruise	Red	Edema	Bruise	Red	Edema
1	1 (M)	(J)	B			<B			–		
2	3 (M)	(J)	<B			–			–		
3	67 (M)	(J)	<B			<B			–		
4	38 (M)		[illegible] B			<B			–		
5	10 (M)	J				<B			<B		
6											
7											
8											
9											
10											
11											
12											
13											
14											
15											
16											
17											
18											
19											
20											
		Total	4	Ø	Ø	4	Ø	Ø	1	Ø	Ø

Br
4JM
1 Adm.

Reaction Description		
B-bruise	R-red	E-edema
B-significant bruise	R-significant redness	E-significant edema
< B-small/diminishing bruise	<R-slight/diminishing redness	<E-diminishing edema
B>-large/increasing bruise	R>-intense/increasing redness	E>-increasing edema

TST Reaction Form

Room: C1 Source: MU Species: Cynos Group#: 12072017

Flashlight: (Yes) / No RE Total # animals in group: 98

			24 hr Reaction			48 hr Reaction			72 hr Reaction		
			Date/Time/Initial 0704 12Dec17 WP			Date/Time/Initial 1030 13Dec17 WP			Date/Time/Initial 1130 14Dec17 WP		
	Cage# (F)	Animal# (J)	Bruise	Red	Edema	Bruise	Red	Edema	Bruise	Red	Edema
1	1 (F)	[illegible] (J)	<B	—	—	<B	—	—	—	—	—
2	5 (F)	[illegible] (J)	—	<R	—	<B	—	—	—	—	—
3	10 (F)	[illegible] (J)	<B	—	—	<B	—	—	—	—	—
4		born (J)	—	—	—	<B	—		—	—	
5											
6											
7											
8			BF			R					
9											
10			3JF			1JF					
11											
12											
13											
14											
15											
16											
17											
18											
19											
20											
		Total	2	1	Ø	4	Ø	Ø	Ø	Ø	Ø

Reaction Description		
B-bruise	R-red	E-edema
B-significant bruise	R-significant redness	E-significant edema
< B-small/diminishing bruise	<R-slight/diminishing redness	<E-diminishing edema
B>-large/increasing bruise	R>-intense/increasing redness	E>-increasing edema

TST Reaction Form

Room: C2 Source: MU Species: Cyno Group#: 12072017

Flashlight: (Yes) / No RE Total # animals in group: 98

			Date/Time/Initial 10Jan18 1605 MP			Date/Time/Initial 11Jan18 1220 MP			Date/Time/Initial 12Jan18 0820 MP		
			24 hr Reaction			48 hr Reaction			72 hr Reaction		
	Cage#	Animal#	Bruise	Red	Edema	Bruise	Red	Edema	Bruise	Red	Edema
1	36 (M)	[illegible] (Adult)	<B	—	—	<B	—		<B		—
2	38 (M)	[illegible] (Adult)	<B	—		—	—		—	—	—
3	40 (M)	[illegible] (Adult)	<B	—		<B	—	—	—	—	
4	206 (M)	[illegible] (J)		—	—	<B	—	—	<B	—	
5											
6											
7											
8											
9											
10											
11											
12											
13											
14											
15											
16											
17											
18											
19											
20											
		Total	3	Ø	Ø	3	Ø	Ø	2	Ø	Ø

BR

1JM
1 Adult M
2 Adult M.

Reaction Description		
B-bruise	R-red	E-edema
B-significant bruise	R-significant redness	E-significant edema
< B-small/diminishing bruise	<R-slight/diminishing redness	<E-diminishing edema
B>-large/increasing bruise	R>-intense/increasing redness	E>-increasing edema

TST Reaction Form

Room: C2 Source: Mu Species: Cynos Group#: 12072017

Flashlight: (Yes) / No RE Total # animals in group: 98

			Date/Time/Initial 0680 12Dec17 MP			Date/Time/Initial 1043 13Dec17 MP			Date/Time/Initial 1142 14Dec17 MP		
			24 hr Reaction			48 hr Reaction			72 hr Reaction		
	Cage#	Animal#	Bruise	Red	Edema	Bruise	Red	Edema	Bruise	Red	Edema
1	1 (M)		(J) <B	—			—			—	
2	4 (M)	both	<B	—		<B	—			—	
3	10 (M)		(J) <B	—		<B	—		<B	—	
4	11 (M)		(J) <B	—		<B	—		<B	—	
5	33 (M)		Adult	R	—		—			—	
6	36 (M)		Adult <B	—		<B	—		<B	—	
7	14 (M)		(J)	—		<·B	—		<B	—	
8	76 (M)		(J)	—		<B	—			—	
9											
10											
11											
12	4		(J)			Br			R		
13			(J)			7JM			1JM.		
14											
15											
16											
17											
18											
19											
20				1 (M) 2 Adult.							
		Total	7	Ø	Ø	6	Ø	Ø	4	Ø	Ø

Reaction Description		
B-bruise	R-red	E-edema
B-significant bruise	R-significant redness	E-significant edema
< B-small/diminishing bruise	<R-slight/diminishing redness	<E-diminishing edema
B>-large/increasing bruise	R>-intense/increasing redness	E>-increasing edema
